# *Syngap^+/−^* CA1 Pyramidal Neurons Exhibit Upregulated Translation of Long MRNAs Associated with LTP

**DOI:** 10.1523/ENEURO.0086-25.2025

**Published:** 2025-05-06

**Authors:** Aditi Singh, Manuela Rizzi, Sang S. Seo, Emily K. Osterweil

**Affiliations:** ^1^Rosamund Stone Zander Translational Neuroscience Center, F. M. Kirby Center, Department of Neurology, Harvard Medical School, Boston Children’s Hospital, Boston, Massachusetts 02115; ^2^Simons Initiative for the Developing Brain, University of Edinburgh, Edinburgh EH8 9XD, United Kingdom; ^3^Centre for Discovery Brain Sciences, University of Edinburgh, Edinburgh EH8 9XD, United Kingdom

**Keywords:** Fragile X, LTD, LTP, Syngap, translation

## Abstract

In the *Syngap^+/−^* model of SYNGAP1-related intellectual disability (SRID), excessive neuronal protein synthesis is linked to deficits in synaptic plasticity. Here, we use Translating Ribosome Affinity Purification and RNA-seq (TRAP-seq) to identify mistranslating mRNAs in *Syngap^+/−^* CA1 pyramidal neurons that exhibit occluded long-term potentiation (LTP). We find the translation environment is significantly altered in a manner that is distinct from the *Fmr1^−/y^* model of fragile X syndrome (FXS), another monogenic model of autism and intellectual disability. The *Syngap^+/−^* translatome is enriched for regulators of DNA repair and mimics changes induced with chemical LTP (cLTP) in WT. This includes a striking upregulation in the translation of mRNAs with a longer-length (>2 kb) coding sequence (CDS). In contrast, long CDS transcripts are downregulated with induction of Gp1 metabotropic glutamate receptor-induced long-term depression (mGluR-LTD) in WT, and in the *Fmr1^−/y^* model that exhibits occluded mGluR-LTD. Together, our results show the *Syngap^+/−^* and *Fmr1^−/y^* models mimic the translation environments of LTP and LTD, respectively, consistent with the saturation of plasticity states in each model. Moreover, we show that translation of >2 kb mRNAs is a defining feature of LTP that is oppositely regulated during LTD, revealing a novel mRNA signature of plasticity.

## Significance Statement

Mutations in *SYNGAP1* result in severe intellectual disability and autism. This study investigates how heterozygous loss of Syngap in mice changes the molecular environment of neurons in the hippocampus, an area important for learning. The results reveal changes in the translating mRNA population of area CA1 that are similar to changes that occur with synaptic strengthening during long-term synaptic potentiation. This suggests that the persistent synaptic strengthening in *Syngap^+/−^* neurons is facilitated by a change in the translating mRNA environment.

## Introduction

New protein synthesis in neurons is required to support experience-dependent learning and is constitutively altered in multiple mouse models of neurodevelopmental disorders (Davis and Squire, 1984; Kelleher and Bear, 2008; Auerbach and Bear, 2010; Holt and Schuman, 2013). Two notable examples of this are fragile X syndrome (FXS) and *SYNGAP1*-related intellectual disability (SRID), which arise from mutations in *FMR1* and *SYNGAP1*, respectively (Qin et al., 2005; Dolen et al., 2007; Osterweil et al., 2010; Wang et al., 2013; Barnes et al., 2015). Both disorders are commonly identified single-gene causes of autism and intellectual disability (ID) that co-occur with other behavioral symptoms including hyperactivity, anxiety, and hypersensitivity to sensory stimuli (Kidd et al., 2014; Mignot et al., 2016). In the *Fmr1^−/y^* model, excessive protein synthesis in the hippocampus facilitates exaggerated long-term synaptic depression downstream of Gp1 mGluR activation (mGluR-LTD; Bear et al., 2004; Dolen et al., 2007; Osterweil et al., 2010). Although excessive hippocampal protein synthesis has also been observed in the *Syngap^+/−^* mouse, much less is known about the role of this change in the observed synaptic plasticity phenotypes.

SynGAP is an essential scaffolding protein that modulates the insertion of AMPA-type glutamate receptors at the postsynaptic density (PSD; Komiyama et al., 2002; Rumbaugh et al., 2006). Along with its role in PSD complexes, SynGAP also contains a GTP activating (GAP) domain that negatively regulates the activity of small GTPases Ras and Rap1 at synapses (Kim et al., 1998; Komiyama et al., 2002; Rumbaugh et al., 2006). The Ras-ERK pathway is a potent regulator of translation, and the excess protein synthesis in *Syngap^+/−^* neurons is corrected with inhibitors of this pathway (Wang et al., 2013; Barnes et al., 2015). A major plasticity deficit observed in *Syngap^+/−^* hippocampus is a significant impairment in LTP induction (Komiyama et al., 2002; Araki et al., 2020, 2024). Reduction in SynGAP expression in mouse or human cultured neurons results in a persistent increase in AMPARs at the PSD and increased dendritic spine size indicative of synaptic strength (Rumbaugh et al., 2006; Llamosas et al., 2020). Extensive in vivo dendritic imaging studies show that SynGAP must be dispersed to allow for insertion of new AMPARs to support LTP (Araki et al., 2015, 2024). Together, these results suggest that there is a persistent synaptic strengthening in *Syngap^+/−^* that occludes induction of LTP (Komiyama et al., 2002; Wang et al., 2013). In addition to this described role in LTP, a study investigating mGluR-LTD in the *Syngap^+/−^* mouse also revealed an exaggeration similar to the *Fmr1^−/y^* model (Barnes et al., 2015).

Here, we sought to understand the role of altered protein synthesis in the hippocampal plasticity phenotypes seen in the *Syngap^+/−^* mouse. To do this, we performed Translating Ribosome Affinity Purification and RNA-seq (TRAP-seq) to profile translating mRNAs in the CA1 pyramidal neurons of *Syngap^+/−^* hippocampus (Heiman et al., 2008). We find dysregulation of a number of transcripts, including a surprising upregulation in those encoding DNA regulatory proteins and chromatin modifiers. We also find that there is little overlap between the differentially expressed transcripts in *Syngap^+/−^* and *Fmr1^−/y^* mutant models. Interestingly, changes seen in the *Syngap^+/−^* TRAP are similar to those induced in WT slices with chemical stimulation of LTP (cLTP), assessed by comparing with previously published CamK2a-ribotag data from cLTP-stimulated hippocampal slices (Chen et al., 2017). In contrast, there is little overlap between *Syngap^+/−^* TRAP and mGluR-LTD TRAP populations. Indeed, a comparison of TRAP-seq datasets from WT stimulated for cLTP or mGluR-LTD reveals a striking divergence in these opposing plasticity states. Gene ontology (GO) and gene set enrichment analysis (GSEA) reveal an increased translation of axonal and cell adhesion effectors during cLTP and a decrease in these factors during mGluR-LTD. In contrast, increased translation of ribosomal and mitochondrial proteins is induced with mGluR-LTD, and these are decreased during cLTP. Further investigation reveals an opposite regulation of translating mRNA populations based on transcript coding sequence (CDS) length. Shorter-length (<1 kb) transcripts encoding metabolic regulators including ribosomal and mitochondrial proteins are reduced, and longer-length (>2 kb) transcripts encoding synaptic and cell adhesion proteins are increased, during cLTP. The opposite length-dependent shift is seen with induction of mGluR-LTD. The same opposite relationship is seen in *Syngap^+/−^* and *Fmr1^−/y^* models. Together, our results show the translating mRNA population in *Syngap^+/−^* CA1 mimics that induced by cLTP in WT, including the upregulation of long mRNAs, which may contribute to the persistent synaptic strengthening and occlusion of LTP in this model.

## Materials and Methods

### Animals

*Syngap^+/−^* mice were originally generated by Komiyama et al. (2002) and were a generous gift from Peter Kind and Seth Grant. These mice were bred using heterozygous crosses and maintained on the C57Black6JOla line (Harlan). CA1-TRAP mice (created by http://gensat.org/ and obtained from Jackson Labs with permission from Nathanial Heintz) were bred on the JAX C57BL/6J background. All experiments were carried out using male littermate mice aged postnatal days (P) 25–32 and studied with the experimenter blind to genotype. While the P25–32 age range may introduce some developmental variability, this window was chosen to capture a stage of robust synaptic plasticity prior to adolescent decline, and all comparisons were performed between littermates closely age-matched within this range to minimize variability. *Syngap^+/−^* and WT littermates were bred from a F1 cross of *Syngap^+/−^* females and CA1-TRAP homozygous males. Mice were group-housed (six maximum) in conventional nonenvironmentally enriched cages with unrestricted food and water access and a 12 h light/dark cycle. Room temperature was maintained at 21 ± 2°C with ambient humidity. Animal husbandry was carried out by University of Edinburgh technical staff. All procedures were performed in accordance with ARRIVE guidelines and the UK Animal Welfare Act, and were approved by the Animal Welfare and Ethical Review Body at the University of Edinburgh.

### TRAP

TRAP was performed on *Syngap^+/−^* CA1-TRAP littermates as described previously in Thomson et al. (2017). Briefly, male littermates (P25–32) were decapitated and hippocampi rapidly dissected in ice-cold PBS. Hippocampi were homogenized in ice-cold lysis buffer (20 mM HEPES, 5 mM MgCl_2_, 150 mM KCl, 0.5 mM DTT, 100 mg/ml cycloheximide, RNase inhibitors and protease inhibitors) using Dounce homogenizers and samples centrifuged at 1,000 × *g* for 10 min to remove large debris. Supernatants were then extracted with 1% NP-40 and 1% DHPC on ice and centrifuged at 20,000 × *g* for 20 min. A 50 ml sample of supernatant was removed for use as Input, and the rest incubated with streptavidin/protein L-coated Dynabeads (Life Technologies) bound to anti-GFP antibodies (HtzGFP-19F7 and HtzGFP-19C8, Memorial Sloan Kettering Centre) overnight at 4°C with gentle mixing. Anti-GFP beads were washed with high salt buffer (20 mM HEPES, 5 mM MgCl_2_, 350 mM KCl, 1% NP-40, 0.5 mM DTT and 100 mg/ml cycloheximide), and RNA was eluted from all samples using Absolutely RNA Nanoprep kit (Agilent) according to the manufacturer’s instructions. RNA yield was quantified using RiboGreen (Life Technologies) and RNA quality was determined by Bioanalyzer analysis.

### RNA-seq library preparation and analysis

RNA with RIN >7 was prepared for RNA-seq using the RNaseq Ovation V2 kit (Nugen), according to manufacturer’s instructions. Samples were sent to Oxford Genomics Centre for sequencing using Illumina HiSeq 2500 or HiSeq 4000. Adapters were removed using cutadapt 2.6 (Martin, 2011) with Python 3.6.3 using these parameters: -j 0 -q 30 -m 50 -a CTGTCTCTTATA -A CTGTCTCTTATA –trim-n. Then, fastqc module (version 0.11.9) was used to analyze the quality of reads. Sequencing reads (50 or 75 bp, paired end) from *Syngap^+/−^*, *Fmr1^−/y^*, cLTP, and mGLUR-LTD datasets were mapped to *Mus musculus* primary assembly (Ensembl release v109) of Mouse Genome GRCm39 using STAR (Spliced Transcripts Alignment to a Reference) RNA-seq aligner v2.7.10b with parameters –outSAMstrandField intronMotif –outFilterIntronMotifs RemoveNoncanonical –outSAMtype BAM SortedByCoordinate. Reads that were uniquely aligned to annotated genes were counted with featureCounts module of subread v2.0.5 (Liao et al., 2014) using default parameters. Differential expression analyses were performed using DESeq2 v1.40.1 (Love et al., 2014) with R version 4.3.0. Lowly expressed genes were filtered out using the criterion that a gene must have at least 10 normalized counts in a minimum of three samples [implemented as rowSums(counts(dds) ≥10) ≥3]. Log_2_ fold change (LFC) shrinkage using the “normal” estimator was applied to reduce the bias toward large fold changes in lowly expressed genes and was used for visualization and gene ranking purposes. The *Syngap^+/−^*, *Fmr1^−/y^*, cLTP, and mGLUR-LTD datasets were analyzed separately. For each comparison, DESeq2 models incorporated sample pairing (matched wild-type and mutant/stimulated animals) using the design_formula = ∼ Pair + Condition. Quality checks were performed on aligned BAM files and read count files using MultiQC v1.10.1 which gives a summary for all quality assessments.

### Gene set enrichment and gene ontology analysis

GSEA v4.3.2 Mac App was downloaded from website (https://www.gsea-msigdb.org/gsea/) and annotated gene sets were used from Molecular Signature Database - MSigDB (v2022.1.Mm). We specifically focused on biological, molecular, and cellular pathways from m5.go.v2022.1, which is a list of ontology gene sets. GSEA analysis was performed using GSEAPreranked method, where genes were ranked by fold change and a “classic” enrichment statistics was used to remove the magnitude bias of ranking metric. Minimum of 20 and maximum of 500 were defined as cutoff for number of genes in a gene set identification with maximum 1,000 permutations. Network plots for GSEA categories were created using igraph_1.4.3 in R version 4.3.0 where the number of shared genes were represented as weights between the two points. GSEA comparison across datasets was performed for significant terms using a cutoff of Nominal *p* values (*p* < 0.01) or FDR (*p*adj < 0.1) as indicated and ranked by normalized enrichment score (NES). Tables summarizing these GSEA categories are supplied as extended data. Gene ontology analysis was performed using ClusterProfileR v4.12 (Yu et al., 2012) and enrichR (Chen et al., 2013). For each analysis only significant terms were selected with a maximum *p* value <0.01 or as indicated.

### Transcript length analysis

CA1 excitatory neuron-specific dataset was retrieved from a publicly available data at GEO GSE74985. Transcript abundance was calculated using RSEM v1.3.0, where rsem-prepare-reference was used to extract reference transcripts and then rsem-calculate-expression to calculate the expression values. The transcript length and other genomic features were obtained from BioMart, and the length of most abundant transcript was used for comparison of CDS length at gene level. Then the transcripts from translating mRNA (TRAP) and total RNA fraction of *Syngap^+/−^*, *FMR1^−/y^*, LTP, and LTD datasets were separated in the bins of >1, 1–2, 2–4, and >4 kb and analyzed for their up- and downregulation.

### Percent overlap estimation

For the percent calculation shown under the Venn diagram overlaps, the percentage was calculated for first mentioned dataset in first Venn circle. The overlap number from this dataset was used as numerator while the selected population including overlap—from total (as indicated with *p* value or *p* adjusted value)—was used as denominator and outcome was multiplied to 100. Thereafter, the percent for similarly and opposite regulation from overlap results was calculated taking the identified set as numerator and overlap as total—denominator.

### Statistical analysis

All statistics were performed using R. For RNA-seq datasets, differential expression was determined using DESeq2 using the default cutoff for significance (adjusted *p* value < 0.1). For GO and GSEA, significance was determined by nominal *p* value and adjusted *p* values with FDR cutoff where indicated. Differences between distributions were compared using two-sample *z* test as indicated.

## Results

### *Syngap^+/−^* and *Fmr1^−/y^* CA1 translatomes are largely dissimilar

**Figure 1.**
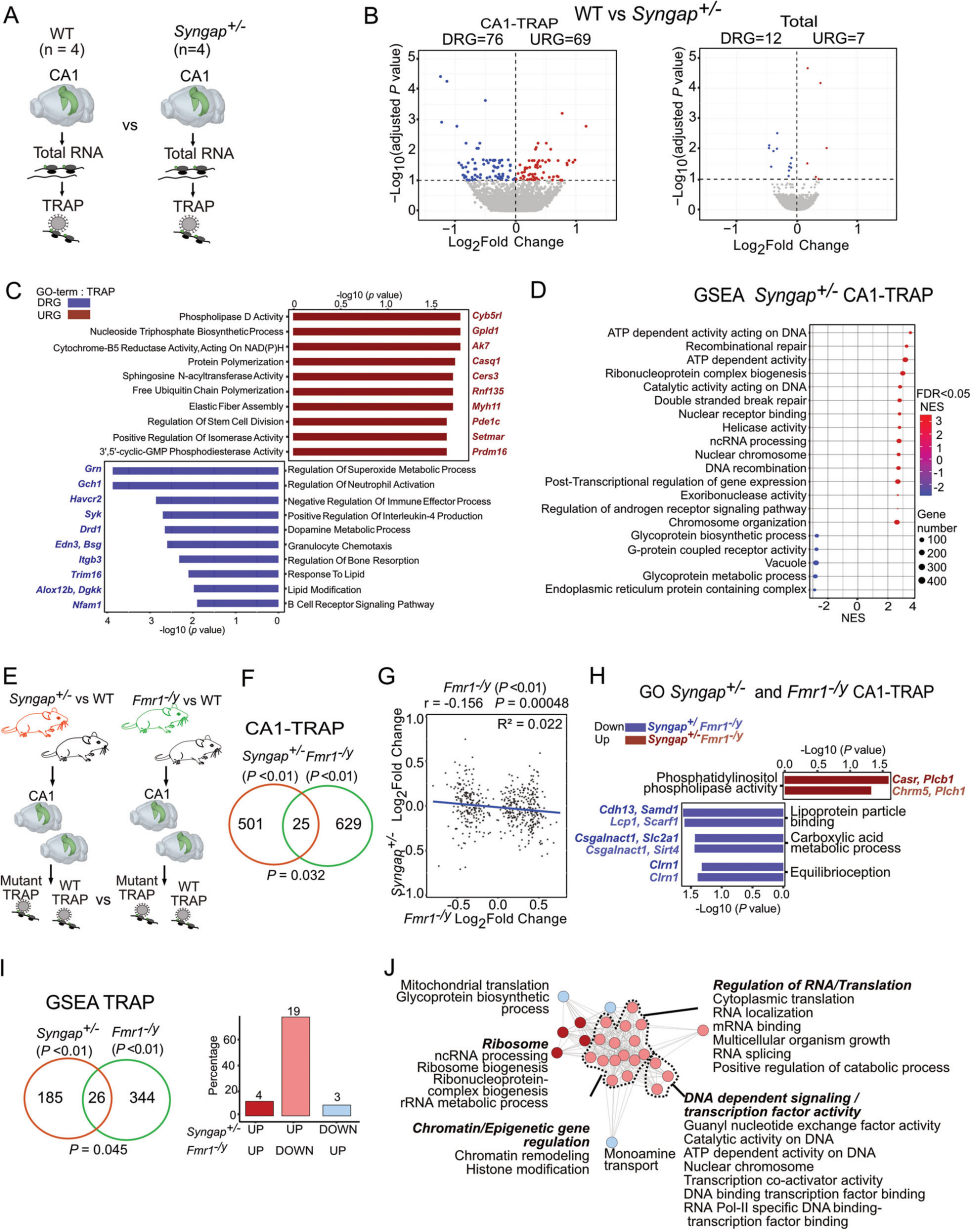
The translating mRNA population in *Syngap^+/−^* CA1 neurons is enriched for DNA repair proteins and is distinct from the population in *Fmr1^−/y^* CA1 neurons. ***A***, Schematic for TRAP-seq and total RNA-seq analysis of *Syngap^+/−^* versus WT (*N* = 4 littermate pairs) from hippocampal CA1 neurons. ***B***, Volcano plots for differential analysis of TRAP-seq data on the left and total RNA-seq on the right show substantial changes in translatome than the transcriptome of *Syngap^+/−^*. Significant transcripts (adjusted *p* value < 0.1) being undertranslated or underexpressed are denoted in blue and overtranslated or overexpressed are denoted in red. ***C***, Gene ontology (GO) analysis of transcripts upregulated and downregulated in translatome between *Syngap^+/−^* versus WT. ***D***, Gene set enrichment analysis (GSEA) of the *Syngap^+/−^* (FDR < 0.05) shows a significant downregulation of glycoprotein metabolism, endoplasmic reticulum, and vacuole-related activities while upregulation of processes related to DNA repair, recombination, including ATP-dependent activity acting on DNA and transcriptional regulation. ***E***, Schematic for TRAP-seq dataset comparison of *Syngap^+/−^* versus WT to the TRAP-seq of *Fmr1^−/y^* versus WT from hippocampal CA1 neurons. ***F***, Quantification of transcripts shows 526 significant transcripts (*p* value < 0.01) are differentially translating in *Syngap^+/−^* and only 25 of those overlap with the significant translatome in *Fmr1^−/y^* (*p = 0.032). ***G***, Transcripts significantly changed in *Fmr1^−/y^* translatome are negatively correlated with *Syngap^+/−^* translatome changes (*r* = −0.156, *R*^2^ = 0.022, *p = 0.00048). ***H***, Gene ontology (GO) analysis of transcripts significantly (*p* value < 0.01) altered in *Syngap^+/^* and *Fmr1^−/y^* shows only few functional processes are regulated similarly in both mutants (*p* value < 0.05). ***I***, To determine whether the gene sets altered in *Syngap^+/−^* are similar to those altered in the *Fmr1^−/y^* translating population, significantly changed gene sets (adjusted *p* value < 0.01) were compared with those significantly changed in the *Fmr1^−/y^* population (adjusted *p* value < 0.01). This reveals a modest overlap of 25 gene sets (*p = 0.045) but 19 of these (85%) are regulated in opposite direction. ***J***, The gene sets inversely modulated between *Syngap^+/−^* and *Fmr1^−/y^* regulate the chromatin remodeling, RNA localization and metabolism, transcription factor, and DNA-dependent activities among others, which are upregulated in *Syngap^+/−^* while downregulated in *Fmr1^−/y^*. Data supported by Extended Data Figures 1-1–1-3 and Tables 1-1–1-7.

The identity of the overly synthesized protein population in *Syngap^+/−^* neurons is not known, and we therefore performed TRAP-seq on *Syngap^+/−^* and WT littermates expressing EGFP-L10a in CA1 pyramidal neurons as in previous work (Fig. 1*A*; Thomson et al., 2017; Seo et al., 2022). Our results show that 145 transcripts are differentially expressed in the *Syngap^+/−^* CA1-TRAP fraction (*p*adj < 0.1; Fig. 1*B*, Extended Data Table 1-1). Of these mRNAs, 69 are upregulated and 76 are downregulated. In contrast to the ribosome-bound TRAP fraction, a comparison of WT and *Syngap^+/−^* total RNA fractions reveals only a small number of differentially expressed transcripts, with seven significantly upregulated and 12 significantly downregulated (*p*adj < 0.1; Fig. 1*B*, Extended Data Table 1-2). The most significantly changed transcripts in the TRAP fraction do not exhibit a similar change in the total RNA fraction, suggesting the effects are not solely due to transcript abundance (Extended Data Fig. 1-1).

10.1523/ENEURO.0086-25.2025.f1-1 Figure 1-1 **Significantly altered transcripts in *Syngap^+/-^* CA1-TRAP are not changed in total transcriptome.** A heatmap of log2foldchanges (L2FC) shows that significant transcripts altered in *Syngap^+/-^* translatome are not changed similarly in the total transcriptome. Download Figure 1-1, TIF file.

**Figure 2.**
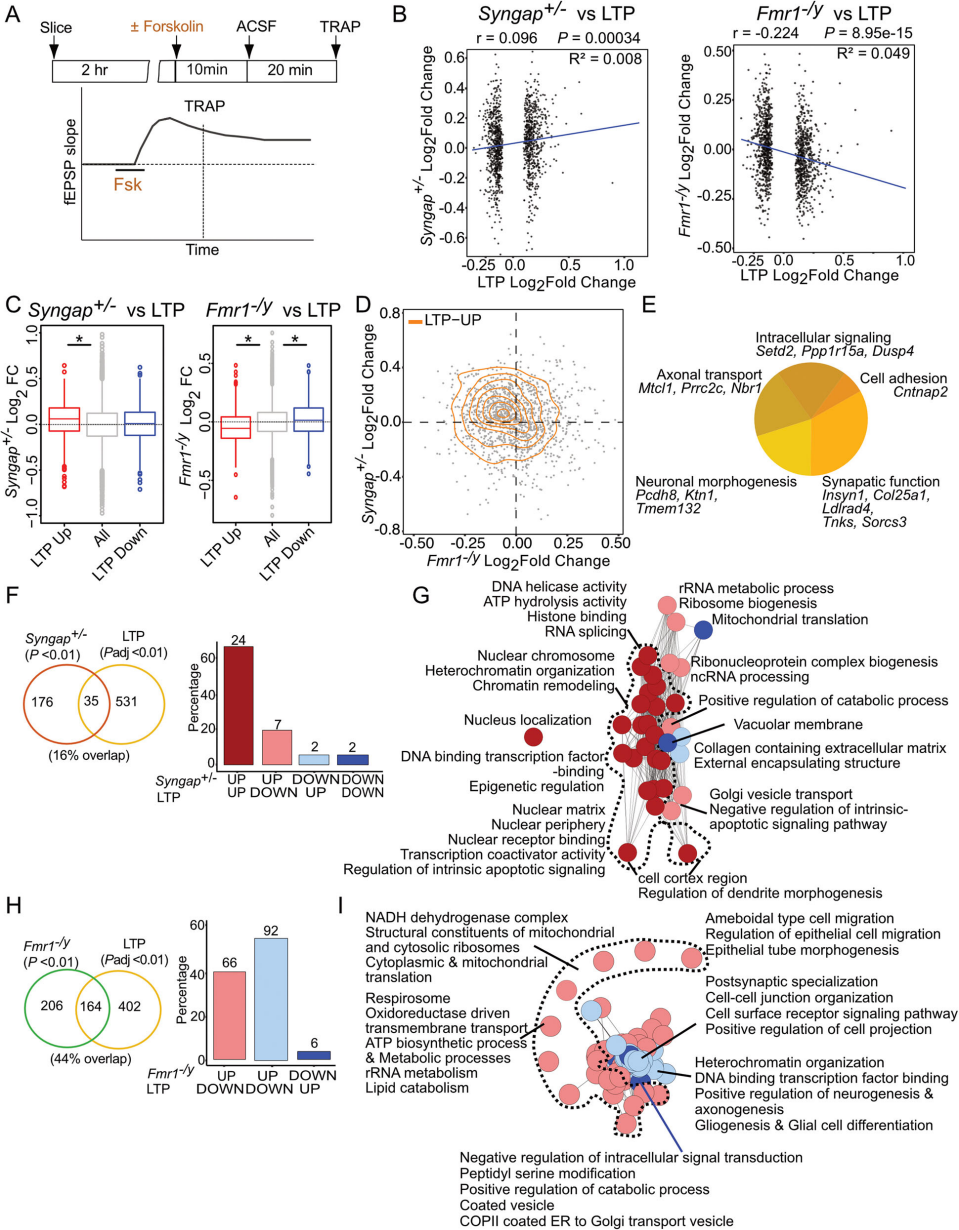
cLTP-specific translation changes in WT match basal changes in *Syngap^+/−^* CA1 neurons, but diverge from changes in *Fmr1^−/y^* CA1 neurons. ***A***, Schematic of the TRAP strategy from wild-type (WT) hippocampal slices stimulated with 50 µM forskolin to induce robust cLTP chemical LTP (Chen et al.) followed by ribotag pulldown and RNA-seq on Camk2a-positive CA1 and CA3 neurons. ***B***, LTP-specific significant transcripts (adjusted *p* value < 0.1) show small but significantly positive correlation with *Syngap^+/−^* translatome (*r* = −0.096, *R*^2^ = 008, *p = 0.00034) while notably negative correlation with *Fmr1^−/y^* translatome changes (*r* = −0.224, *R*^2^ = 0.049, *p = 8.95 × 10^−15^). ***C***, Analysis of the LTP-specific significant transcripts in the *Syngap^+/−^* translatome shows significant increase in LTP-upregulated transcripts but no change in LTP-downregulated transcript (Kruskal–Wallis test *p = 2.15 × 10^−12^, post hoc two-sided Wilcoxon rank-sum test up *p = 2.96 × 10^−13^, down *p* = 0.055), while LTP-specific significant transcripts in the *Fmr1^−/y^* translatome show significant opposing change in both groups—LTP-upregulated and LTP-downregulated transcripts (Kruskal–Wallis test *p < 2.2 × 10^−16^, post hoc two-sided Wilcoxon rank-sum test up *p < 2.2 × 10^−16^, down *p = 0.00078). Boxplots display the distribution of Log_2_FoldChange values across LTP-upregulated and LTP-downregulated group of transcripts. The box represents the interquartile range (25th–75th percentile), the center line indicates the median, and whiskers extend to 1.5 times the interquartile range. Data beyond the whiskers are shown as outliers. ***D***, Joint distribution analysis of LTP-specific transcripts between *Syngap^+/−^* and *Fmr1^−/y^* translatomes in a 2D density plot shows the positive distribution pattern of LTP-upregulated transcripts in *Syngap^+/−^*. ***E***, Analysis of the significantly upregulated LTP-specific transcript population that are also upregulated in *Syngap^+/−^* translatome fraction identifies transcripts, which are involved in synaptic functions, intracellular signaling, neuronal morphogenesis, axonal transport, and cell adhesion. ***F***, To determine whether the gene sets altered in *Syngap^+/−^* are also altered in the cLTP translating population, significantly changed *Syngap^+/−^* gene sets (*p* value < 0.01) were compared with those significantly changed in the cLTP population (adjusted *p* value < 0.01). This unveils an overlap of 35 gene sets (*p* = 0.054); nonetheless, majority of these (74.28%) are similarly upregulated in both. ***G***, The gene sets that are alike and upregulated in both *Syngap^+/−^* and cLTP are involved in dendrite morphogenesis, chromosome organization, transcription, and DNA-dependent regulatory activities among others. ***H***, Comparison of the gene sets altered in cLTP (adjusted *p* value < 0.01) with the ones altered in *Fmr1^−/y^* (*p* value < 0.01) shows a greater overlap of 164 (44.3%) terms (*p = 1.24 × 10^−57^); however, 56% of these terms are changed in an opposite direction. ***I***, Shared gene sets between cLTP and *Fmr1^−/y^* regulate important processes such as mitochondrial function, different metabolic processes, and translation which is upregulated in *Fmr1^−/y^* while downregulated with cLTP. The gene sets involved in axonogenesis, synaptic adhesion, postsynaptic specialization, and heterochromatin organization are upregulated with LTP but downregulated in *Fmr1^−/y^*. Data supported by Extended Data Figures 2-1 and 2-2 and Tables 2-1–2-3.

10.1523/ENEURO.0086-25.2025.f1-2 Figure 1-2 **Mistranslation of distinct transcripts in *Syngap^+/^* and *Fmr1^-/y^*.** Quantification of transcripts shows 145 significant transcripts (*P*adj < 0.1) are differentially translating in *Syngap^+/-^* and only 1 of those overlap with the significant translatome in *Fmr1^-/y^ P* = 0.052). Download Figure 1-2, TIF file.

**Figure 3.**
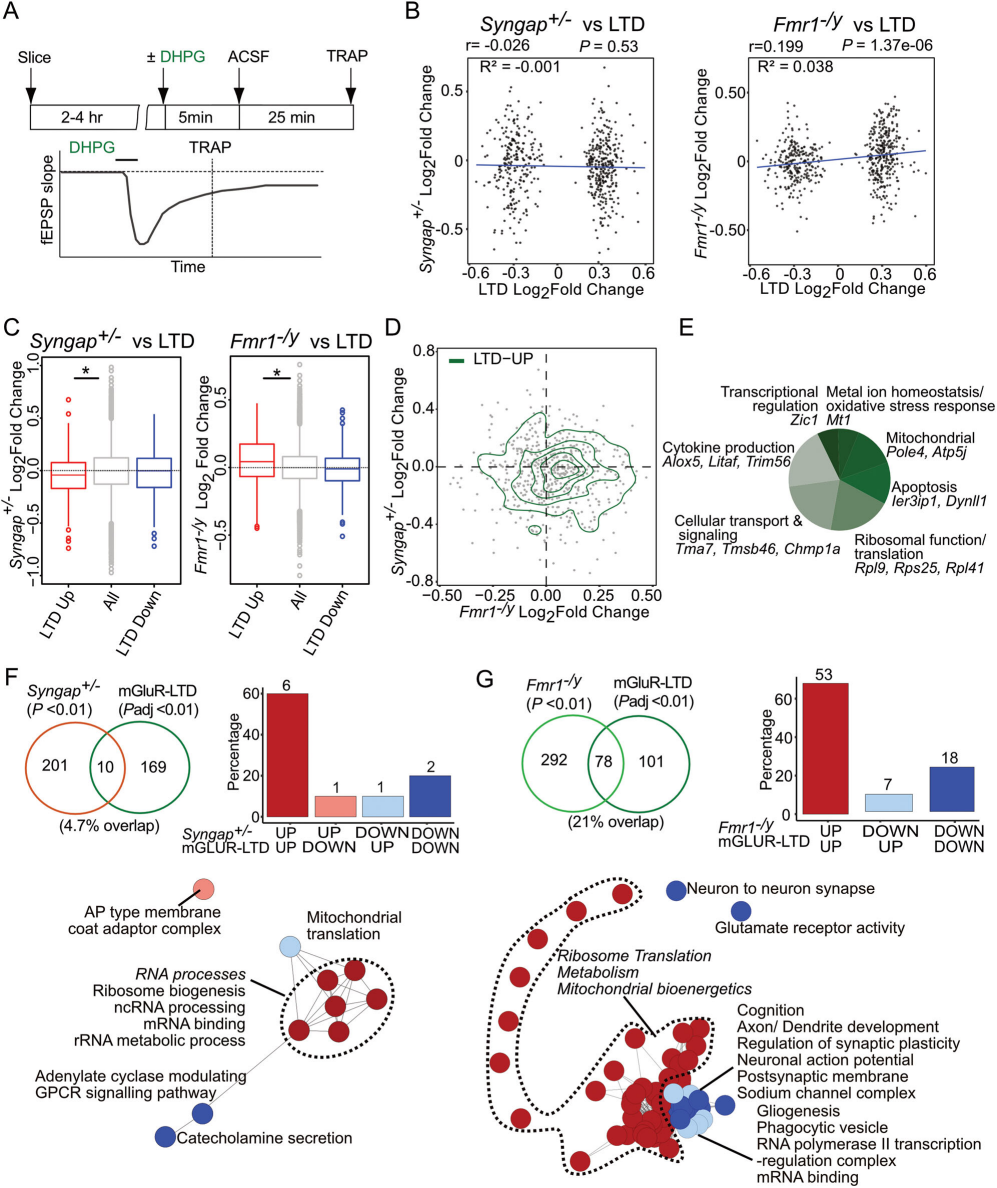
mGluR-LTD–specific translation changes in CA1 neurons match basal changes *Fmr1^−/y^* but not *Syngap^+/−^* hippocampus. ***A***, Schematic of the TRAP strategy from wild-type (WT) hippocampal slices stimulated with a 5 min pulse of 50 µM S-DHPG that induces robust mGluR-LTD (Seo et al.) followed by TRAP-seq on CA1 pyramidal neurons. ***B***, LTD-specific significant transcripts (adjusted *p* value < 0.1) show no correlation with *Syngap^+/−^* translatome (*r* = −0.026, *R*^2^ = −0.001, *p* = 0.533) while remarkably significant positive correlation with *Fmr1^−/y^* translatome changes (*r* = 0.199, *R*^2^ = 0.038 *p = 1.375 × 10^−06^). ***C***, Analysis of the LTD-specific significant transcripts in the *Syngap^+/−^* translatome shows significant decrease in LTD- upregulated transcripts (Kruskal–Wallis test *p = 0.0001, post hoc two-sided Wilcoxon rank-sum test up *p = 2.74 × 10^−05^, down *p* = 0.07). In contrast, LTD-specific significant transcripts in the *Fmr1^−/y^* translatome show notably significant increase in LTD-upregulated transcripts (Kruskal–Wallis test *p = 2.66 × 10^−08^, post hoc two-sided Wilcoxon rank-sum test up *p = 7.04 × 10^−09^, down *p* = 0.057). Boxplots display the distribution of Log_2_FoldChange values across LTD-upregulated and LTD-downregulated group of transcripts. The box represents the interquartile range (25th–75th percentile), the center line indicates the median, and whiskers extend to 1.5 times the interquartile range. Data beyond the whiskers are shown as outliers. ***D***, Combined distribution analysis of LTD-specific transcripts between *Syngap^+/−^* and *Fmr1^−/y^* translatomes in a 2D density plot shows the positive distribution pattern of LTD-upregulated transcripts with *Fmr1^−/y^* translation changes. ***E***, Analysis of the significantly upregulated LTD-specific transcript population that are also upregulated in *Fmr1^−/y^* translatome fraction identifies transcripts which are involved in apoptosis, ribosomal as well as mitochondrial functions, transcription regulation and cellular transport. ***F***, Comparison of the gene sets significantly altered in *Syngap^+/−^* (*p* value < 0.01) with the ones altered in LTD (adjusted *p* value < 0.01) reveals nonsignificant overlap of merely 4.7% (*p* = 0.3927527). ***G***, To determine whether the gene sets altered in mGluR-LTD translating population match *Fmr1^−/y^* translatome, significantly changed *Fmr1^−/y^* gene sets (*p* value < 0.01) were compared with those significantly changed in the LTD population (adjusted *p* value < 0.01). This unveils a greater overlap of 21% with 78 terms (*p = 1.83 × 10^−46^) and 91% of these terms are changed in similar direction. The gene sets that are upregulated alike in LTD and *Fmr1^−/y^* are involved in ribosome and mitochondrial function, while similarly downregulated sets are related to neuronal activity and synaptic membrane functions. Data supported by Extended Data Figures 3-1 and 3-2 and Tables 3-1, 3-2.

10.1523/ENEURO.0086-25.2025.f1-3 Figure 1-3 **Non-linear modeling of the correlation between *Syngap^+/-^* and *Fmr1^-/y^* CA1-TRAP Log_2_ fold changes.** This plot shows the relationship between transcripts significantly changed in the *Fmr1^-/y^* dataset (*P* < 0.01) and their corresponding fold changes in the *Syngap^+/-^* dataset. Both linear regression (blue dashed line) and Generalized Additive Model (GAM, red curve) fits are shown, with substantial overlap indicating that the relationship is largely captured by a linear model. Download Figure 1-3, TIF file.

10.1523/ENEURO.0086-25.2025.t1-1 Table 1-1 ***Syngap^+/-^* CA1 TRAP-seq.** DESeq2 results for all transcripts in TRAP-seq from hippocampal CA1 in *Syngap^+/-^* and WT littermates. Download Table 1-1, XLSX file.

10.1523/ENEURO.0086-25.2025.t1-2 Table 1-2 ***Syngap^+/-^* total hippocampal transcriptome.** DESeq2 results for all transcripts in total hippocampal fraction from *Syngap^+/-^* and WT littermates. Download Table 1-2, XLSX file.

10.1523/ENEURO.0086-25.2025.t1-3 Table 1-3 **GO analysis *Syngap^+/-^* CA1 TRAP-seq.** Gene ontology analysis terms for *Syngap^+/-^* CA1-TRAP significantly Up- and Down-regulated transcripts (*P*adj < 0.1) highlights nucleotide biosynthesis, protein modification and changes related to cellular resilience. Download Table 1-3, XLSX file.

10.1523/ENEURO.0086-25.2025.t1-4 Table 1-4 **GSEA of *Syngap^+/-^* CA1 TRAP-seq.** GSEA on CA1-TRAP transcript population from *Syngap^+/-^* versus WT shows enriched gene sets related to DNA modification and recombinational repair. Download Table 1-4, XLSX file.

10.1523/ENEURO.0086-25.2025.t1-5 Table 1-5 ***Syngap^+/-^* versus *Fmr1^-/y^* CA1-TRAP**. DEseq2 analysis of significantly differentially regulated transcripts in CA1-TRAP of *Syngap^+/-^* vs WT and *Fmr1^-/y^* vs WT. Download Table 1-5, XLSX file.

10.1523/ENEURO.0086-25.2025.t1-6 Table 1-6 **GO analysis *Syngap^+/-^* versus *Fmr1^-/y^* CA1-TRAP.** Gene ontology analysis terms for *Syngap*^+/-^ CA1-TRAP significantly Up- and Down-regulated transcripts (*P* < 0.01) highlights DNA repair and shows minimal overlap with *Fmr1^-/y^* CA1-TRAP (GO terms. Download Table 1-6, XLSX file.

10.1523/ENEURO.0086-25.2025.t1-7 Table 1-7 **GSEA of *Syngap^+/-^* versus *Fmr1^-/y^* CA1 TRAP-seq.** GSEA on the CA1-TRAP transcript population from *Syngap^+/-^* vs WT and *Fmr1^-/y^* vs WT reveals an overlapping population. Download Table 1-7, XLSX file.

To understand how the differentially expressed transcripts alter molecular and cellular process in *Syngap^+/−^*, we performed a GO analysis on the most significantly up- and downregulated populations (*p*adj < 0.1). This revealed an upregulation in categories related to cytochrome-B5 reductase activity [acting on NAD(P)H], nucleoside triphosphate biosynthetic process, and phospholipase D activity which suggests an increase in cellular functions associated with energy metabolism, nucleotide synthesis, and neuronal membrane remodeling. Additionally, the enrichment of positive regulation of isomerase activity, free ubiquitin chain polymerization, sphingosine N-acyltransferase activity, and protein polymerization points to increased protein modification and elongation, potentially contributing to the observed phenotype of altered protein synthesis (Fig. 1*C*, Extended Data Table 1-3). The downregulated population is enriched for immune-related signaling pathways, dopamine metabolic process, and regulation of lipid modification suggesting a reduction in neurotransmitter metabolism. To complement these findings and gain a comprehensive understanding of functional enrichment—capturing gene sets with subtle, coordinated changes rather than focusing solely on the most significantly altered genes—we next performed GSEA on the CA1-TRAP population. This showed upregulation of processes related to DNA modification, including recombinational repair and ATP-dependent activity (led by *Mcm2 and Mcm4* core enrichment), as well as RNA regulatory terms suggesting increased genomic stability and transcriptional regulation demands in *Syngap*^+/−^ neurons (Fig. 1*D*, Extended Data Table 1-4). In contrast, the most significantly downregulated gene sets are related to endoplasmic reticulum, glycoprotein metabolism, and vacuoles. In neurons, these pathways are critical for protein folding, trafficking, and synaptic signaling. Together, these changes highlight a potential trade-off in *Syngap*^+/−^ neurons, with increased investment in DNA repair and stability at the expense of glycoprotein metabolism and receptor activity. This imbalance may impact cellular resilience, signaling efficiency, and ultimately synaptic function, potentially contributing to the impaired cognitive and behavioral phenotypes.

Both *Syngap^+/−^* and *Fmr1^−/y^* mice are models of autism and ID; however, they express different plasticity phenotypes in hippocampal CA1. In the *Fmr1^−/y^* mouse, a basal elevation of protein synthesis occludes further translation downstream of mGluRs, resulting in an exaggeration of mGluR-LTD that no longer requires protein synthesis (Huber et al., 2002; Hou et al., 2006; Nosyreva and Huber, 2006; Dolen et al., 2007; Osterweil et al., 2010). Although an increase in mGluR-LTD is seen in the *Syngap^+/−^* hippocampus, there is also a robust deficit in LTP that is due to occlusion; i.e., synaptic strengthening processes are already saturated, preventing further LTP induction. To investigate whether similar differences were seen in the translating mRNA populations, we compared *Syngap^+/−^* CA1-TRAP with *Fmr1^−/y^* mice bred to the same CA1-TRAP line (Fig. 1*E*; Thomson et al., 2017; Seo et al., 2022). A comparison between *Syngap^+/−^* and *Fmr1^−/y^* CA1-TRAP populations (*p*adj < 0.1) is not significant (*p* = 0.052) with only one common transcript (Extended Data Fig. 1-2). Since we are comparing two distinct TRAP datasets, we used a slightly relaxed threshold (*p* < 0.01), which reveals a small but significant 4.75% overlap of differentially expressed transcripts (*p = 0.032); however, only 15 mRNAs are dysregulated in the same direction (Fig. 1*F*, Extended Data Table 1-5). Additionally, a correlation between significantly altered transcripts in the *Fmr1^−/y^* CA1-TRAP with the expression of these genes in *Syngap^+/−^* CA1-TRAP reveals a small but significant negative correlation (*p = 0.00048, *r* = −0.16, *R*^2^ = 0.022; Fig. 1*G*). The data showed a linear trend, and nonlinear models using generalized additive models (GAMs) closely overlapped with the linear regression line (Extended Data Fig. 1-3), suggesting that the relationship is adequately captured by a linear model. Furthermore, even with a relaxed threshold (*p* value < 0.05), the GO analysis of significantly altered transcripts in the *Fmr1^−/y^* CA1-TRAP shows minimal overlap with the GO terms identified in *Syngap^+/−^* (also at *p* value < 0.05). The only overlapping terms are related to membrane remodeling, metabolism, and equilibrioception (Fig. 1*H*, Extended Data Table 1-6). This indicates a lack of similarity between the two mutant models.

To assess whether there was any similarity in the gene sets altered in *Syngap^+/−^* and *Fmr1^−/y^* mutants, we compared GSEA results from the TRAP fractions from each mutant. Our results show that there is a 12% significant overlap (*p = 0.045; Fig. 1*I*, Extended Data Table 1-7). However, the vast majority of overlapping terms (84.6%) are changed in the opposite direction. The terms shared in both mutants relate to chromatin, transcription, DNA activity, and RNA splicing, which are upregulated in the *Syngap^+/−^* CA1-TRAP and downregulated in the *Fmr1^−/y^* CA1-TRAP (Fig. 1*J*). Together, these results suggest that shared changes in the *Syngap^+/−^* and *Fmr1^−/y^* models are mostly opposing.

### *Syngap^+/−^* but not *Fmr1^−/y^* CA1-TRAP shows changes consistent with cLTP

In previous study, TRAP-seq was performed on CA1 pyramidal neurons in acute hippocampal slices 30 min after application of a 5 min pulse of 50 µM S-DHPG, which induces robust mGluR-LTD (Seo et al., 2022). We compared these changes with those basally altered in *Fmr1^−/y^* CA1-TRAP and found a similarity that suggests a saturation of LTD-related protein synthesis. Given the opposite profiles seen between *Syngap^+/−^* and *Fmr1^−/y^* CA1-TRAP populations, and the saturation of LTP in *Syngap^+/−^* CA1, we wondered whether the mistranslating population in *Syngap^+/−^* would be similar to that of LTP induction in WT. To investigate this, we analyzed a dataset generated by Chen et al., who performed ribotag pulldown and RNA-seq on Camk2a-positive CA1 and CA3 neurons in acute hippocampal slices 30 min poststimulation with 50 µM forskolin to induce robust cLTP (Fig. 2*A*; Chen et al., 2017). The RNA-seq reads for LTP datasets were remapped to the current Ensemble mouse genome and processing approach similar to *Syngap^+/−^* and *Fmr1^−/y^* datasets (see Materials and Methods). Then, control versus stimulated populations were compared using DESeq2 with parameters identical to all datasets (*p*adj < 0.1).

10.1523/ENEURO.0086-25.2025.f2-1 Figure 2-1 **Non-linear modeling of the relationship between LTP-induced transcript changes and CA1-TRAP profiles in *Syngap^+/-^* and *Fmr1^-/y^* mice.** Scatterplots show the Log2 fold changes of transcripts significantly altered in the LTP dataset (*P* < 0.01) plotted against their corresponding fold changes in *Syngap^+/-^* (left) and *Fmr1^-/y^* (right) CA1-TRAP datasets. Linear regression fits (blue dashed lines) and Generalized Additive Model (GAM) fits (red curves) are overlaid. In the *Fmr1^-/y^* comparison, a substantial overlap between the GAM and linear fits, indicates a predominantly linear inverse relationship. In contrast, the *Syngap^+/-^* comparison showed the deviation of the GAM curve from the linear trend suggests a non-uniform relationship, consistent with heterogeneous upregulation of LTP-induced genes. Download Figure 2-1, TIF file.

10.1523/ENEURO.0086-25.2025.f2-2 Figure 2-2 **Comaprison of cLTP and mGLUR-LTD related translation changes in WT with *Syngap^+/-^* and *Fmr1^-/y^* CA1 neurons. (A)** Analysis of the LTP significant transcripts in the *Syngap^+/-^* translatome shows significant increase in LTP- upregulated transcripts but no change in LTP- downregulated transcript (Kruskal-Wallis test *P = 3.30e-13, Post hoc two-sided Wilcoxon rank-sum test up *P = 4.77e-14, down *P* = 0.97), while LTP significant transcripts in the *Fmr1^-/y^* translatome show significant opposing change in both groups- LTP- upregulated and LTP- downregulated transcripts (Kruskal-Wallis test *P < 2.2e-16, Post hoc two-sided Wilcoxon rank-sum test up *P < 2.2e-16, down *P = 0.00013). **(B)** Analysis of the LTD significant transcripts in the *Syngap^+/-^* translatome shows significant decrease in LTD- upregulated transcripts (Kruskal-Wallis test *P = 9.81e-05, Post hoc two-sided Wilcoxon rank-sum test up *P = 2.29e-05, down *P* = 0.67). In contrast, LTD significant transcripts in the *Fmr1^-/y^* translatome show notably significant increase in LTD-upregulated transcripts (Kruskal-Wallis test *P =1.587e-11, Post hoc two-sided Wilcoxon rank-sum test up *P =7.71e-11, down *P = 0.0026). Boxplots display the distribution of Log_2_FoldChange values across LTP/LTD - up and down - regulated group of transcripts. The box represents the interquartile range (25^th^ - 75^th^ percentile), the center line indicates the median, and whiskers extend to 1.5 times the interquartile range. Data beyond the whiskers are shown as outliers. Download Figure 2-2, TIF file.

10.1523/ENEURO.0086-25.2025.t2-1 Table 2-1 **Common transcripts between LTP and LTD datasets.** Common transcripts between DEseq2 of Camk2a- ribotag transcripts from hippocampal slices at 30  min post-forskolin treatment (versus unstimulated) and DEseq2 of CA1-TRAP transcripts from hippocampal slices at 30  min post-DHPG treatment (versus unstimulated). These were assumed to be the changes that occur with general plasticity stimulation. Download Table 2-1, XLSX file.

10.1523/ENEURO.0086-25.2025.t2-2 Table 2-2 **LTP-specific changes in Camk2a-ribotag population (*P*adj < 0.1)**. DEseq2 of Camk2a- ribotag transcripts from hippocampal slices at 30  min post-forskolin treatment (versus unstimulated). Download Table 2-2, XLSX file.

10.1523/ENEURO.0086-25.2025.t2-3 Table 2-3 **GSEA comparison between *Syngap^+/-^*** /**WT, LTP/unstimulated, and *Fmr1^-/y^*/WT.** Comparison of gene sets changed in *Syngap^+/-^* TRAP, LTP ribotag, and *Fmr1^-/y^* TRAP. Download Table 2-3, XLSX file.

To rule out changes that occur with general plasticity stimulation, we removed transcripts that were also significantly changed in LTD dataset (Extended Data Table 2-1), resulting in a “LTP-specific” population. Our results show that there is a significant positive correlation between LTP-induced transcripts and those changed in *Syngap^+/−^* CA1-TRAP (*p = 0.00034, *r* = 0.096, *R*^2^ = 0.008; Fig. 2*B*, Extended Data Table 2-2). In contrast, a similar comparison to the *Fmr1^−/y^* revealed that transcripts upregulated with LTP were significantly negatively correlated (*p = 8.95 × 10^−15^, *r* = −0.224, *R*^2^ = 0.049). While the correlation between LTP and *Fmr1^−/y^* appeared largely linear, as supported by overlapping GAM and linear regression fits, the relationship in *Syngap^+/−^* showed a nonlinear pattern, with the GAM curve deviating from the linear trend (Extended Data Fig. 2-1). This suggests that only a subset of LTP-induced genes may be upregulated in *Syngap^+/−^* neurons, reflecting a more complex or heterogeneous shift toward an LTP-like state. To ask whether significantly up- and downregulated populations might be distinctly changed in the *Syngap^+/−^* or *Fmr1^−/y^* populations, we performed a separate analysis to individually compare these groups (Fig. 2*C*). Our results show a significant increase in LTP-upregulated transcripts in the *Syngap^+/−^* (*p = 2.96 × 10^−13^), but no significant downregulation in the LTP-downregulated population (*p* = 0.055). In *Fmr1^−/y^*, there is a significant opposing change observed in both groups (up *p < 2.2 × 10^−16^, down *p = 0.00078; Fig. 2*C*). Furthermore, this trend was consistent across all transcripts that were significantly altered in LTP, not limited to specific populations (Extended Data Fig. 2-2). These results suggest that the differential translating mRNAs in *Syngap^+/−^* CA1 show similar changes during cLTP. The most significantly upregulated transcripts in LTP that are also upregulated in *Syngap^+/−^* include regulators of synaptic function and plasticity (*Insyn1*, *Col25a1*, *Ldlrad4*, *Tnks*, *Sorcs3*), neuronal morphogenesis (*Pcdh8*, *Ktn1*, *Tmem132*), axonal transport (*Mtcl1*, *Prrc2c*, *Nbr1*), intracellular signaling (*Setd2*, *Ppp1r15a*, *Dusp4*), and cell adhesion (*Cntnap2*; Fig. 2*D*,*E*).

Next, we used GSEA to compare gene sets significantly changed in *Syngap^+/−^* CA1-TRAP with those changed during cLTP. Our results show that 17% of gene sets changed in the *Syngap^+/−^* population are also changed with cLTP in WT (*p* = 0.054; Fig. 2*F*, Extended Data Table 2-3). Importantly, most of the overlapping terms are shifted in the same direction (74.28%). The majority of similarly upregulated categories include those involved in chromosome organization, transcription, and DNA regulation (Fig. 2*G*). In stark contrast, a comparison between LTP and *Fmr1^−/y^* populations shows a 44.3% overlap (*p = 1.24 × 10^−57^); however, a remarkable 56% of these terms are changed in an opposite direction (Fig. 2*H*). The overlapping categories upregulated in *Fmr1^−/y^* and downregulated with LTP include those involved in mitochondrial function and ribosomes (Fig. 2*I*). Categories upregulated with LTP and downregulated in *Fmr1^−/y^* include those involved in synaptic adhesion and heterochromatin organization. These results indicate that changes to the translating mRNA population of hippocampal pyramidal neurons induced by cLTP are similar to basal changes in *Syngap^+/−^* hippocampus and opposite to basal changes in *Fmr1^−/y^* hippocampus.

### *Fmr1^−/y^* but not *Syngap^+/−^* CA1 pyramidal neurons show translation changes similar to those induced with mGluR-LTD

Despite the basal synaptic strengthening and LTP occlusion seen in *Syngap^+/−^* hippocampus and cortex, a previous study also revealed an exaggeration of hippocampal mGluR-LTD in in this model (Komiyama et al., 2002; Barnes et al., 2015; Araki et al., 2024). We therefore compared the expression profile seen in CA1-TRAP isolated from WT slices 30 min after stimulation of mGluR-LTD (published in Seo et al., 2022, reprocessed with identical parameters—see Materials and Methods) to the profile seen in *Syngap^+/−^* CA1-TRAP (Fig. 3*A*). Transcripts overlapping with the LTP dataset were removed, resulting in a “LTD-specific” population. We find no correlation between transcripts changed with LTD and those changed in *Syngap^+/−^* neurons (*p* = 0.53, *r* = −0.026, *R*^2^ = −0.001; Fig. 3*B*, Extended Data Table 3-1). This contrasts with the small but significant positive correlation between LTD and *Fmr1^−/y^* populations (*p = 1.37 × 10^−06^, *r* = 0.199, *R*^2^ = 0.038). Nonlinear smoothing using GAMs confirmed these trends, as the GAM fits closely overlapped with the corresponding linear regression lines. A comparison of significantly up- and downregulated populations shows a small but significant downregulation of transcripts upregulated with LTD in the *Syngap^+/−^* population (*p = 2.741 × 10^−05^) and no change in the LTD-downregulated population (Fig. 3*C*). In contrast, the LTD up- and downregulated populations are changed in a similar direction in the *Fmr1^−/y^* pool (up *p = 7.049 × 10^−09^, down *p* = 0.05777; Fig. 3*C*,*D*). The trends from LTD-specific transcripts are replicated even for total significantly altered transcripts *Syngap^+/−^* and *Fmr1^−/y^* (Extended Data Fig. 2-1). A functional breakdown of mRNAs significantly upregulated with LTD that are also upregulated in *Fmr1^−/y^* highlights those involved in ribosome function (*Rpl9*, *Rps25*, *Rpl41*), cellular transport (*Tma7*, *Tmsb46*, *Chmp1a*), cytokine production (*Alox5*, *Litaf*, *Trim56*), mitochondria (*Pole4*, *Atp5j*), apoptosis (*Ier3ip1*, *Dynll1*), oxidative stress (*Mt1*), and transcription (*Zic1*; Fig. 3*E*).

10.1523/ENEURO.0086-25.2025.f3-1 Figure 3-1 **Non-linear modeling of the relationship between LTD-induced transcript changes and CA1-TRAP profiles in *Syngap^+/-^* and *Fmr1^-/y^* mice.** Scatterplots show the log2 fold changes of transcripts significantly altered in the LTD dataset (*P* < 0.01) plotted against their corresponding fold changes in *Syngap^+/-^* (left) and *Fmr1^-/**y**^* (right) CA1-TRAP datasets. Linear regression fits (blue dashed lines) and Generalized Additive Model (GAM) fits (red curves) are overlaid. In the *Fmr1^-/**y**^* comparison, a small but significant positive correlation is observed, with close overlap between the GAM and linear fits, indicating a largely linear relationship. In contrast, no correlation was observed in the *Syngap^+/-^* dataset, and slight deviation of the GAM curve from the linear trend suggests a non-uniform or flat relationship between LTD-regulated genes and their expression in Syngap+/- neurons. Download Figure 3-1, TIF file.

10.1523/ENEURO.0086-25.2025.t3-1 Table 3-1 **LTD-specific changes in CA1-TRAP population (*P*adj < 0.1).** DEseq2 of CA1-TRAP transcripts from hippocampal slices at 30  min post-DHPG treatment (versus unstimulated). Download Table 3-1, XLSX file.

10.1523/ENEURO.0086-25.2025.t3-2 Table 3-2 GSEA comparison between *Syngap^+/-^* /WT, LTD/unstimulated, and *Fmr1^-/y^* /WT. Comparison of gene sets changed in *Syngap^+/-^* TRAP, LTD TRAP, and *Fmr1^-/y^* TRAP. Download Table 3-2, XLSX file.

Next, to investigate any similarities in functional groups, we compared gene set enrichment in LTD and *Syngap^+/−^* populations. This revealed a small 4.7% overlap between *Syngap^+/−^* and LTD populations, with only eight changed in a similar direction (*p* = 0.39; Fig. 3*F*, Extended Data Table 3-2). In contrast, a comparison between LTD and *Fmr1^−/y^* reveals a 21% overlap with 78 gene sets shifted in the same direction (*p = 1.83 × 10^−46^; Fig. 3*G*). The similarly upregulated gene sets are those relating to ribosome and mitochondrial function, and similarly downregulated sets are related to neuronal and synaptic function. These results suggest that the translation profile induced with LTD is similar to the basal population in *Fmr1^−/y^* but not *Syngap^+/−^* CA1.

### The mRNA populations translated during LTP and LTD are largely divergent

**Figure 4.**
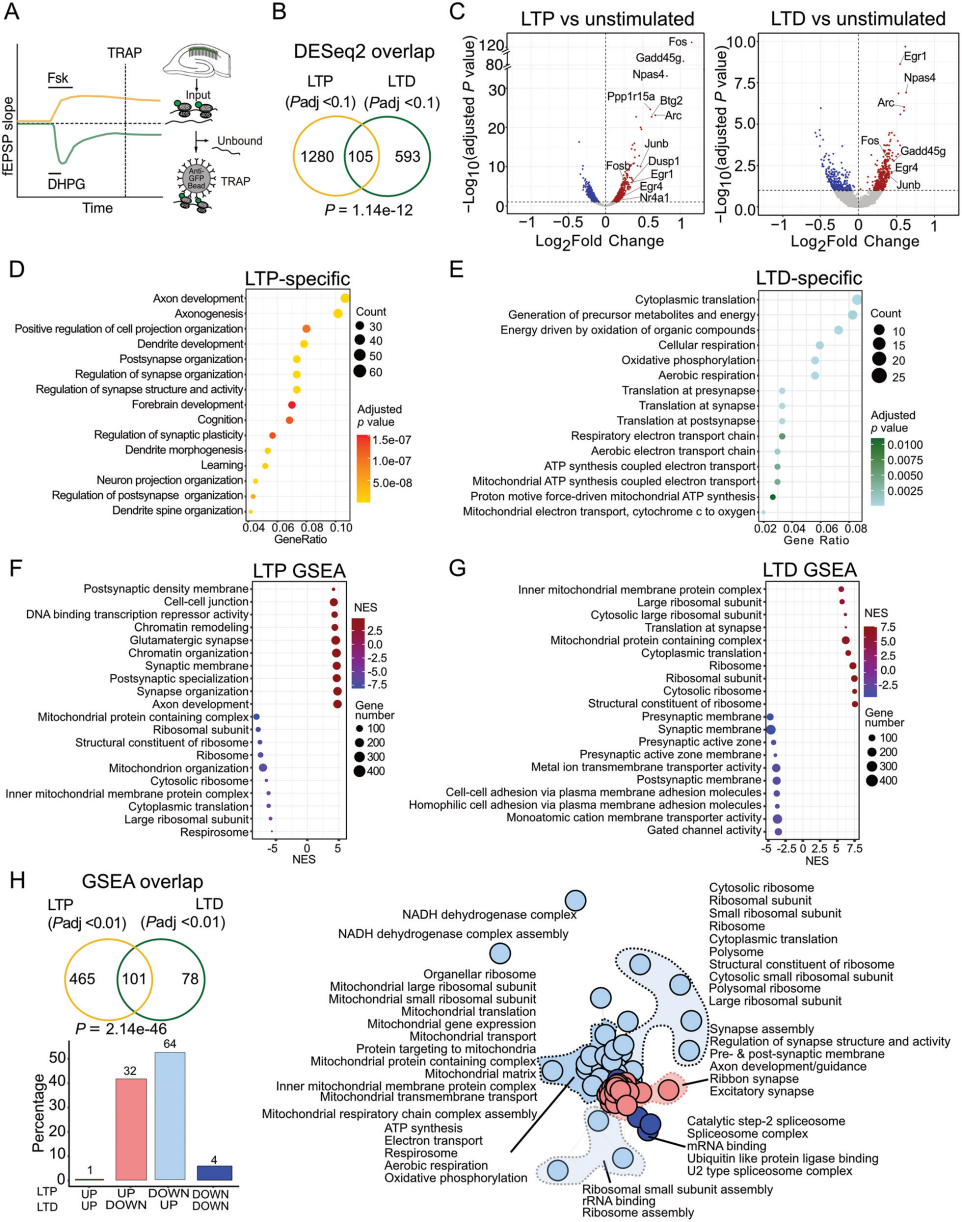
LTP and LTD induce distinct translatome shifts including opposite changes in synaptic transcripts. ***A***, Schematic of the TRAP strategy from wild-type (WT) mouse hippocampal slices induced for mGluR-LTD (Sang et al.) and chemical LTP (Chen et al.). ***B***, LTP and LTD comparison shows distinctly translated transcripts in both phenomena. Differential analysis was performed to identify synaptic plasticity related transcripts, i.e., LTP versus unstimulated control and LTD versus unstimulated control. LTP and LTD significant (adjusted *p* value < 0.1) transcripts were compared to find a common overlap and specific population of transcripts. ***C***, Volcano plot of ribosome-bound translating population of transcripts in LTP and LTD. Significant transcripts (adjusted *p* value  < 0.1) being undertranslated are denoted in blue and overtranslated are denoted in red. Both LTP and LTD stimulation exhibit upregulated translation of immediate early genes such as *Arc*, *Fos*, *Npas4*, and *Egr1* indicating neuronal activation. LTP-specific translation of *Ppp1r15a*, *Btg2*, and *Nr4a* among other transcripts is also remarkable. ***D***, ***E***, Gene ontology analysis of LTP- and LTD-specific transcripts shows their unique functions. LTP-specific transcripts predominantly regulate axonogenesis, cell projection, and dendrite development processes among others (***D***) while LTD-specific transcripts primarily regulate cytoplasmic translation, pre- and postsynaptic translation, metabolism, and energy precursors synthesis (***E***). ***F***, GSEA analysis of the LTP versus unstimulated control TRAP-seq dataset identified over translation of gene sets related to synapse organization, cell–cell junction, and chromatin remodeling in LTP (adjusted *p* value < 0.1). The downregulated gene sets in LTP are involved in mitochondrial and ribosomal functions as well as cytoplasmic translation. ***G***, GSEA analysis of the LTD versus unstimulated control TRAP-seq dataset identified over translation of gene sets related to ribosome, translation, and mitochondrial terms in LTD (adjusted *p* value < 0.1), while the downregulated gene sets are involved in pre- and postsynaptic membrane functions as well as cell–cell adhesion. ***H***, To determine uniquely altered gene sets altered, a comparison of significant (adjusted *p* value < 0.1) gene sets identified by GSEA in both LTP and LTD TRAP-seq datasets was performed. This reveals a common pool of 101 gene sets (*p = 2.14 × 10^−46^) and their remarkably opposite regulation between LTP versus LTD. The gene sets involved in synapse assembly, axon development, and regulation of synapse structure are upregulated in LTP but downregulated in LTD. The gene sets related to cytoplasmic and mitochondrial ribosome, ATP synthesis, and electron transport among other significant terms are downregulated in LTP but upregulated in LTD. Data supported by Extended Data Figure 4-1 and Tables 4-1–4-3.

Our *Syngap^+/−^* comparisons led us to the realization that there are significant differences between the translating mRNA populations induced with LTP versus LTD in hippocampal neurons. Although there have been many differential expression studies examining the differences in gene expression between LTP and LTD, we are not aware of a direct comparison between translation profiles in CA1 pyramidal neurons. We therefore compared these populations (Fig. 4*A*). Interestingly, a comparison of significantly changed transcripts revealed most were stimulation-specific (1,280 LTP, 593 LTD); however, a significant overlap of 105 transcripts was also observed (*p = 1.14 × 10^−12^; Fig. 4*B*, Extended Data Table 4-1). Many of the shared transcripts upregulated in both stimulations are immediate early genes (IEGs; Fig. 4*C*) that mark neuronal activation (i.e., *Fos*, *Npas4*, *Junb*, etc.; Greenberg et al., 1986; Cole et al., 1989). To assess the functional relevance of transcripts significantly upregulated with cLTP, we performed GO analyses (Fig. 4*D*, Extended Data Table 4-2. This shows an enrichment in axon development, dendrite development, and synapse structure. In contrast, the population significantly upregulated with induction of mGluR-LTD is enriched for cytoplasmic translation, respiration, and metabolism (Fig. 4*E*). These results indicate the transcripts translated during cLTP and those translated during mGluR-LTD encode for very different functional classes. The shared population upregulated by both stimulations is enriched for transcription factors and regulators of membrane excitability including IEGs, while the shared downregulated population is enriched for calcium regulators and GTPases (Extended Data Fig. 4-1). This is consistent with a general cellular response to stimulation.

10.1523/ENEURO.0086-25.2025.f4-1 Figure 4-1 **Gene ontology analysis of commonly changed transcripts in LTP and LTD.** GO terms enriched in the overtranslated populations in both LTP & LTD regulate membrane depolarization, DNA binding while the commonly undertranslated transcripts primarily regulate calcium ion sequestration, GTPase activity, nucleotide biosynthesis. Download Figure 4-1, TIF file.

10.1523/ENEURO.0086-25.2025.t4-1 Table 4-1 Comparison between LTP- and LTD-specific changes (*P*adj < 0.1). Commonly changed transcripts in both LTP and LTD datasets. Download Table 4-1, XLSX file.

10.1523/ENEURO.0086-25.2025.t4-2 Table 4-2 **GO analyses of significantly upregulated transcripts in LTP and LTD datasets.** GO analyses of significantly upregulated transcripts in LTP-specific and LTD-specific datasets. Download Table 4-2, XLSX file.

10.1523/ENEURO.0086-25.2025.t4-3 Table 4-3 **GSEA comparison between LTP/unstimulated and LTD/unstimulated datasets**. Comparison of GSEA enriched terms from LTP and LTD datasets. Download Table 4-3, XLSX file.

To assess whether the gene sets shifted with cLTP and mGluR-LTD were similarly divergent, we performed GSEA on each population. Our analysis of LTP-specific changes revealed an upregulation of synaptic terms and transcription/chromatin regulators and a downregulation of ribosome- and mitochondria-related terms (Fig. 4*F*, Extended Data Table 4-3). Conversely, LTD-specific changes include an upregulation of ribosome- and mitochondria-related terms and a downregulation of synaptic terms (Fig. 4*G*). Interestingly, a comparison of gene sets shifted with each stimulation showed a significant overlap (101 shared, 465 LTP, 78 LTD; *p = 2.146771 × 10^−46^), but a striking divergence in the direction of change within shared categories (Fig. 4*H*). Specifically, LTP is defined by a significant upregulation in synaptic stability transcripts and a downregulation in ribosomal and mitochondrial transcripts. In contrast, LTD is defined as an upregulation in ribosomal/mitochondrial transcripts and a downregulation in synaptic stability gene sets. Together, these results provide compelling evidence that the mRNAs translated to support LTP and LTD are divergent, and a subset is oppositely regulated.

### Long transcripts encoding synaptic structural components are bidirectionally translated with LTP and LTD in hippocampal pyramidal neurons

In previous work, a negative correlation was observed between differential expression and transcript length in the translating population of *Fmr1^−/y^* CA1 pyramidal neurons (Seo et al., 2022). This can be seen as a significant increase in shorter mRNAs within the population (i.e., <1 kb) and a significant downregulation of the longer transcripts (i.e., >2 kb). Interestingly, there is a functional segregation of effectors encoded by genes of differing length within the neuronal genome that is also seen in the transcriptome (Gabel et al., 2015; Zylka et al., 2015; McCoy et al., 2018). In particular, shorter genes encode ribosomal proteins, mitochondrial proteins, nucleosome proteins, and regulators of metabolic function. In contrast, longer genes encode proteins involved in cell adhesion, ion channels, and cytoskeleton proteins. This effect can be seen in total transcript length but also in the length of the CDS that does not include untranslated regions (UTRs). We hypothesize the length-dependent shift in the neuronal translatome of *Fmr1^−/y^* neurons is contributing to the constitutive underproduction of synaptic stability proteins (Seo et al., 2022).

**Figure 5.**
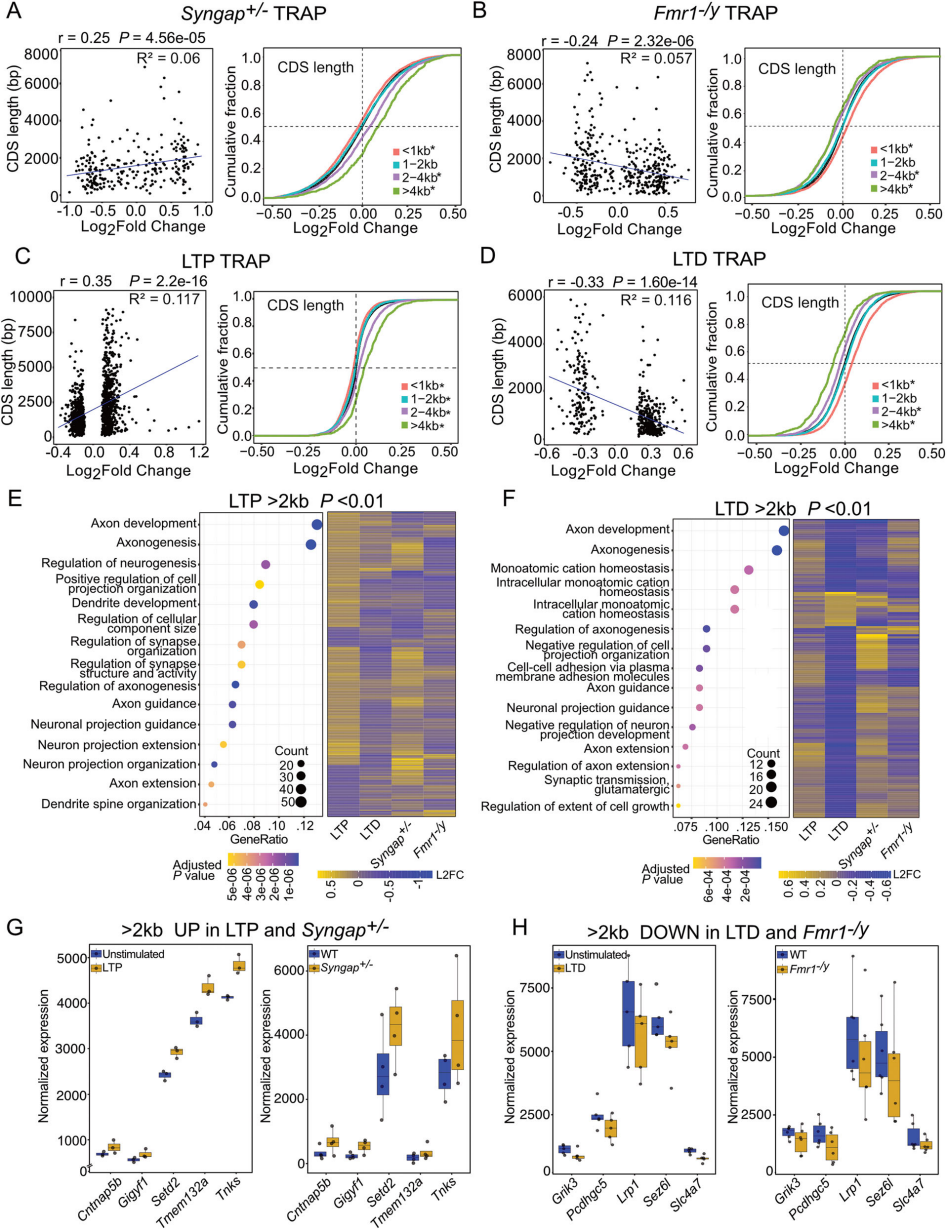
Translation of long (>2 kb) transcripts is bidirectionally altered by stimulation of LTP versus LTD, and this is mimicked in *Syngap^+/−^* and *Fmr1^−/y^* mutant CA1 neurons. ***A***, Transcripts significantly changed in *Syngap^+/−^* translatome (*p* value < 0.01) show significant positive correlation with longer CDS length (left, *r* = 0.25, *R*^2^ = 0.06, *p = 4.56 × 10^−05^). A binned analysis on CDS lengths of altered translatome shows that *Syngap^+/−^* TRAP fraction exhibits upregulated translation of longer transcripts (two-sample *z* test; >4 kb vs all: *z* = 10.716, *p < 2.2 × 10^−16^, 2–4 kb vs all: *z* = 7.8933, *p = 2.94 × 10^−15^, 1–2 kb vs all: *z* = −0.92171, *p* = 0.35, <1 kb vs all: *z* = −7.6739, *p = 1.66 × 10^−14^). ***B***, Transcripts significantly changed in *Fmr1^−/y^* translatome (*p* value < 0.01) show significant negative correlation with longer CDS length (left, *r* = −0.24, *R*^2^ = 0.057, *p = 2.32 × 10^−06^). A binned analysis on CDS lengths of altered translatome shows that *Fmr1^−/y^* TRAP fraction exhibits decreased translation of longer transcripts (two-sample *z* test; >4 kb vs all: *z* = −7.5046, *p = 6.16 × 10^−14^, 2–4 kb vs all: *z* = −10.079, *p < 2.2 × 10^−16^, 1–2 kb vs all: *z* = −1.5061, *p* = 0.132, <1 kb vs all: *z* = 12.301, *p < 2.2 × 10^−16^). ***C***, Analysis of cLTP translatome (*p* value < 0.01) shows significant positive correlation with longer CDS length (left, *r* = 0.35, *R*^2^ = 0.117, *p = 2.2 × 10^−16^). A binned analysis on CDS lengths of altered translatome shows that cLTP causes increased translation of longer transcripts (two-sample *z* test; >4 kb vs all: *z* = 13.987, *p < 2.2 × 10^−16^, 2–4 kb vs all: *z* = 10.401, *p < 2.2 × 10^−16^, 1–2 kb vs all: *z* = −4.9587, *p = 7.09 × 10^−07^, <1 kb vs all: *z* = −13.1, *p < 2.2 × 10^−16^). ***D***, Analysis of mGluR-LTD translatome (*p* value < 0.01) shows significant negative correlation with longer CDS length (left, *r* = −0.33, *R*^2^ = 0.116, *p = 1.60 × 10^−14^). A binned analysis on CDS lengths of altered translatome in LTD exhibits decreased translation of longer transcripts (two-sample *z* test; >4 kb vs all: *z* = −14.638, *p < 2.2 × 10^−16^, 2–4 kb vs all: *z* = −13.203, *p < 2.2 × 10^−16^, 1–2 kb vs all: *z* = 1.2064, *p* = 0.22, < 1 kb vs all: *z* = 21.175, *p < 2.2 × 10^−16^). ***E***, Gene ontology analysis of long transcripts (CDS length >2 kb) in LTP shows their functions in axon development, cell projection, and synapse structure organization (left). These transcripts are upregulated (log_2_foldchanges, L2FC >0) and show largely similar alteration with *Syngap^+/−^* while opposite patterns of alteration (L2FC <0) in LTD as well as *Fmr1^−/y^* (right). ***F***, Gene ontology analysis of long transcripts (CDS length >2 kb) in LTD shows they are related to axon development, cell projection, and synapse organization (left) similar to LTP but translation of these transcripts is decreased in LTD and *Fmr1^−/y^* opposite to LTP and *Syngap^+/−^* (right). ***G***, ***H***, Bidirectionally altered long transcripts in LTP and *Syngap^+/−^* (***G***) versus LTD and *Fmr1^−/y^* (H). Data supported by Extended Data Figures 5-1 and 5-2 and Tables 5-1–5-3.

To examine whether a similar length-dependent translation shift is present in *Syngap^+/−^* CA1 neurons, we compared differential expression to CDS length in the significantly changed population (*p* < 0.01). Our results show a significant positive correlation between expression and transcript CDS length in the *Syngap^+/−^* TRAP-seq population (*p = 4.56 × 10^−05^, *r* = 0.253, *R*^2^ = 0.06; Fig. 5*A*, Extended Data Fig 5-1*A*). Further analysis shows a significant increase in the length of the upregulated population that does not extend to total transcript length, 3′UTR length, or 5′UTR (Fig. 5-2*A*, Extended Data Table 5-1). To assess whether this effect can be seen in the entire population, we compared the differential expression of all transcripts binned by CDS length (<1 kb, 1–2  kb, 2–4 kb, >4 kb) as compared with the average population. Consistent with our correlation analysis, we find a positive length shift in the *Syngap^+/−^* translatome that can be seen as a reduction in <1 kb (*p = 1.669 × 10^−14^) and an increase in 2–4 kb (*p = 2.943 × 10^−15^) and >4 kb (*p < 2.2 × 10^−16^). As shown previously, this length shift is negative in the *Fmr1^−/y^* TRAP-seq population (Fig. 5*B*, Extended Data Fig. 5-1*B*).

10.1523/ENEURO.0086-25.2025.t5-1 Table 5-1 **Genomic features for *Syngap^+/-^, Fmr1^-/y^*, LTP, and LTD datasets.** List of Transcript length, CDS length, 3’UTR length and 5’UTR length of differentially expressed transcripts from *Syngap^+/-^*, *Fmr1^-/y^*, LTP and LTD datasets. Download Table 5-1, XLSX file.

10.1523/ENEURO.0086-25.2025.f5-1 Figure 5-1 **Non-linear modeling of the relationship between CDS length and transcript regulation in *Syngap^+/-^* and *Fmr1^-/y^* CA1-TRAP datasets and WT cLTP/LTD datasets.** Scatterplots show the relationship between transcript coding sequence (CDS) length and log2 fold change in: (A) *Syngap^+/-^* CA1-TRAP vs LTP-induced genes, (B) *Fmr1^-/y^* CA1-TRAP vs LTD-induced genes, (C) WT CA1-TRAP following cLTP stimulation, and (D) WT CA1-TRAP following mGluR-LTD. Each panel displays a linear regression fit (blue dashed line) and a Generalized Additive Model (GAM) fit (red curve). In the WT cLTP and *Syngap^+/-^* datasets, longer CDS length was positively associated with transcript upregulation, though the GAM fits in *Syngap^+/-^* suggest a complex non-linear trend with peak upregulation observed in a subset of transcripts under ∼2500 bp. Conversely, in LTD and *Fmr1^-/y^* datasets, CDS length showed a negative correlation with transcript expression while the GAM fit indicate subtle deviations from linearity. Download Figure 5-1, TIF file.

10.1523/ENEURO.0086-25.2025.f5-2 Figure 5-2 **Genomic features analysis of significant transcripts (P value < 0.01) in *Syngap^+/-^* and cLTP datasets. (A)** Analysis of the transcript length in *Syngap^+/-^* translatome (Kruskal-Wallis test *P* = 0.52); Analysis of the CDS length in *Syngap^+/-^* translatome (Kruskal-Wallis test *P = 0.0001, Post hoc two-sided Wilcoxon rank-sum test up *P = 3.80e-05, down *P* = 0.05); Analysis of the 5’ UTR length in *Syngap^+/-^* translatome (Kruskal-Wallis test *P* = 0.6362); Analysis of the 3’ UTR length in *Syngap^+/-^* translatome (Kruskal-Wallis test *P* = 0.88). **(B)** Analysis of the transcript length in LTP translatome (Kruskal-Wallis test *P < 2.2e-16, Post hoc two- sided Wilcoxon rank-sum test up *P < 2.2e-16, down *P = 0.017); Analysis of the CDS length in LTP translatome (Kruskal-Wallis test *P < 2.2e-16, Post hoc two-sided Wilcoxon rank-sum test up *P < 2.2e- 16, down *P = 0.002); Analysis of the 5’ UTR length in LTP translatome (Kruskal-Wallis test *P < 2.2e-16, Post hoc two-sided Wilcoxon rank-sum test up *P < 2.2e-16, down *P* = 0.15); Analysis of the 3’ UTR length in LTP translatome (Kruskal-Wallis test *P = 3.38e-09, Post hoc two-sided Wilcoxon rank-sum test up *P = 5.97e-10, down *P = 0.04). Boxplots display the distribution of Log_2_ base pairs (bp) for transcript length or cds length and bp or Kilo-bp values of UTR lengths across Up and Down - regulated group of transcripts. The box represents the interquartile range (25^th^ - 75^th^ percentile), the center line indicates the median, and whiskers extend to 1.5 times the interquartile range. Data beyond the whiskers are shown as outliers. Download Figure 5-2, TIF file.

10.1523/ENEURO.0086-25.2025.t5-2 Table 5-2 GO analyses of >2  kb CDS transcripts significantly changed (*P* < 0.01) in LTP and LTD datasets. Significant GO terms enriched in significantly changed >2Kb CDS transcripts from LTP and LTD datasets. Download Table 5-2, XLSX file.

10.1523/ENEURO.0086-25.2025.t5-3 Table 5-3 **Convergent >2  kb CDS transcript changes in *Syngap^+/-^* and LTP, and in *Fmr1^-/y^* and LTD populations**. List of >2  kb CDS transcripts significantly upregulated in both *Syngap^+/-^* and LTP populations, and significantly downregulated in both *Fmr1^-/y^* and LTD populations. Download Table 5-3, XLSX file.

As the *Syngap^+/−^* population exhibits changes consistent with LTP, we next investigated whether a length-dependent shift exists in the WT population stimulated for cLTP. Our results reveal a striking positive correlation between transcript CDS length and differential expression in the WT ribotag population stimulated for cLTP (*p = 2.2 × 10^−16^, *r* = 0.35, *R*^2^ = 0.117; Fig. 5*C*, Extended Data Fig. 5-1*C*). Nonlinear smoothing using GAMs supports an overall positive relationship between CDS length and transcript upregulation in the cLTP condition, consistent with the linear trend. However, the distribution is not uniform—several of the most highly upregulated transcripts have CDS lengths below 2,500 bp, and a subset of longer transcripts (>2,500 bp) are downregulated, indicating heterogeneity within the length-dependent response. As reported in Chen et al., a significant increase in 3′UTR length is also seen in the upregulated population, along with an increase in total transcript length and 5′UTR length (Fig. 5-2*B*, Extended Data Table 5-2). The CDS length shift is also observed in a binned analysis across the translatome (<1 kb *p < 2.2 × 10^−16^, 1–2 kb *p = 7.096 × 10^−07^, 2–4 kb *p < 2.2 × 10^−16^, >4 kb *p < 2.2 × 10^−16^). The same analysis of the LTD population reveals a negative correlation (as previously published; Fig. 5*D*, Extended Data Fig. 5-1*D*). Together, these results show a positive length-dependent translation shift in *Syngap^+/−^* CA1 neurons that matches that seen with induction of cLTP in WT. This stands in stark contrast to the negative length shift seen in *Fmr1^−/y^* CA1 neurons and in those induced for mGluR-LTD.

Given the similar upregulation of longer (>2 kb) mRNAs in the *Syngap^+/−^* population and in the LTP-induced population, we wondered whether we could observe a profile consistent with persistent synaptic strength in this population. To evaluate this, we performed a GO analysis of the significantly changed transcripts >2 kb in the LTP dataset, the majority of which are upregulated (76%, 321/421; Fig. 5*E*, Extended Data Table 5-2). This revealed a clear enrichment in axon and dendrite development as well as synaptic structure, consistent with synaptic strengthening. A heatmap comparing these 421 transcripts shows both the opposing expression in LTD and *Fmr1^−/y^* populations and a similar expression in the *Syngap^+/−^* population. Next, we investigated the >2 kb transcripts significantly changed with mGluR-LTD, the majority of which are downregulated (86.8%, 138/159; Fig. 5*F*). GO analysis shows that this population is similarly enriched for terms related to axon development and synaptic structure, consistent with a profile for synaptic weakening. A heatmap comparing these 159 transcripts shows both the opposing expression in LTP and *Syngap^+/−^* populations and a similar expression in the *Fmr1^−/y^* population.

Collectively, our results suggest a model whereby increased translation of long (> 2kb) mRNAs in hippocampal pyramidal neurons, many of which are constitutively upregulated in *Syngap^+/−^* CA1, supports synaptic strengthening. In contrast, reduced translation of long mRNAs, which are constitutively undertranslating in *Fmr1^−/y^*, supports synaptic weakening. We therefore identified the most significantly upregulated >2 kb transcripts in both *Syngap^+/−^* and LTP populations: *Gigyf1*, *Cntnap5b*, *Tnks*, *Setd2*, and *Tmem132a* (Fig. 5*G*, Extended Data Table 5-3). *Gigyf1* regulates ERK signaling, a pathway linked to synaptic plasticity and implicated in autism. *Cntnap5b* supports cell adhesion and synapse formation, critical for neural connectivity, with links to autism and ID. *Tnks* aids structural integrity and neuronal morphology via Wnt signaling, a pathway altered in ASD. *Setd2*, a histone methyltransferase with mutations associated with ID. *Tmem132a* influences ER stress and cellular adhesion. Transcripts most significantly downregulated in both *Fmr1^−/y^* and mGluR-LTD populations include *Pcdhgc5*, *Sez6l*, *Lrp1*, *Grik3*, and *Slc4a7* (Fig. 5*H*, Extended Data Table 5-3). *Pcdhgc5* maintains synaptic specificity; *Sez6l* is essential for ER functions, mutations identified in ASD; *Lrp1* is critical for neurotransmitter receptor recycling, impacting learning and memory; *Grik3* modulates excitatory signaling, affecting the excitatory–inhibitory balance in ASD; and *Slc4a7* maintains ion transport and neuronal pH, supporting cellular excitability (Extended Data Table 5-3).

Together, these up- and downregulated targets, majority of which are linked to autism and ID, underscore distinct molecular mechanisms through which *Syngap*^+/−^ and *Fmr1*^−/y^ mouse models show impaired neurodevelopment, synaptic function, and cognitive outcomes.

## Discussion

This study sought to identify differentially translating mRNAs in CA1 pyramidal neurons of the *Syngap^+/−^* hippocampus that might participate in synaptic phenotypes. We find that there is a significant increase in DNA regulators and a correlation with changes induced with cLTP in WT. This is opposite to the translating population seen in *Fmr1^−/y^* CA1, where there is an increase in ribosomal proteins and changes that mimic induction of mGluR-LTD in WT. Interestingly, we also find that cLTP induces a translation profile that is strikingly different from that induced with mGluR-LTD. This includes an increase in the translation of longer mRNAs >2 kb, a profile that matches basal changes in the *Syngap^+/−^* population. In contrast, long mRNAs are decreased with induction of LTD in WT and basally downregulated in the *Fmr1^−/y^* population. The >2 kb transcripts significantly upregulated upon induction of LTP and downregulated upon induction of LTD encode regulators of axon/dendrite stability and synaptic adhesion. Overlapping the population changed with plasticity with the populations changed in *Syngap^+/−^* or *Fmr1^−/y^* CA1-TRAP identifies a common list of candidates for the occlusion of LTP or LTD in these models.

There are limitations to this study. First, although we use TRAP as a proxy for the translating mRNA population, it is important to note that this population represents a combined measurement of RNA abundance and ribosome association. This means that changes we observe cannot be attributed to translation alone and may be due to either an increase in ribosome engagement or a change in the availability of mRNA. However, we note the same length-dependent change in *Fmr1^−/y^* neurons is seen in ribosome profiling experiments from fly and mouse models where transcript abundance is not a cofactor (Greenblatt and Spradling, 2018; Aryal et al., 2021). Furthermore, TRAP-seq does not measure protein levels directly, and our conclusions about translational regulation should be interpreted as reflecting—potential rather than confirmed—changes in protein synthesis. Given the technical challenges of obtaining high-resolution, cell-type-specific proteomic data from hippocampal neurons in vivo, TRAP remains a powerful and widely used tool for profiling translational regulation at cell-type resolution. Nonetheless, future studies incorporating complementary proteomic approaches would help to directly validate protein-level changes in these models. Another limitation is that the paradigms used to stimulate LTP and LTD in hippocampal slices are chemical agonists of PKA or Gp1 mGluRs, which likely have many effects beyond those relevant to synaptic plasticity (Chen et al., 2017; Seo et al., 2022). Although we cannot know which changes are directly responsible for the change in synaptic strength, we note that there is a similar increase in IEGs that mark neuronal activity in both stimulations (Fig. 4*C*). This suggests that the opposing changes we observe are not likely due to a change in overall cellular activity. In addition, while *Syngap^+/−^* and *Fmr1^−/y^* TRAP-seq datasets include tissue spanning both dorsal and ventral hippocampus, dorsal CA1 is known to exhibit more robust translational responses to synaptic plasticity paradigms. Thus, we expect that dorsal signals contribute substantially to the observed patterns in our data, supporting the validity of comparison with cLTP and LTD datasets that predominantly sample from dorsal hippocampus. Although the cLTP dataset includes transcripts from both CA3 and CA1 regions, the high translational activity in CA1 and common focus of LTP paradigms on this region suggest that CA1 contributes significantly to the observed signal.

Although there have been several studies of translation differences in mouse models of FXS, relatively few have been performed in *Syngap^+/−^* models. Here, we identify a unique translation signature that could support persistent changes in neuronal function and plasticity. Indeed, our TRAP-seq results reveal an increased nucleotide enrichment of gene sets including minichromosome maintenance (MCM) subunits *Mcm2* and *Mcm4* within the *Syngap^+/−^* population (Fig. 1*D*). The MCM complex is a collection of DNA helicases that are essential for unwinding DNA during replication (Mei and Cook, 2021; Yadav and Polasek-Sedlackova, 2024). The association of MCM with DNA maintains genome integrity during cell-cycle progression, and it is essential for controlling the speed of replication (Sedlackova et al., 2020; Yadav and Polasek-Sedlackova, 2024). The increase of MCM subunits in the *Syngap^+/−^* TRAP is curious and suggests a potential mechanism for the precocious neurogenesis phenotype that has been observed in organoids cultured from SYNGAP1 haploinsufficiency patients (Birtele et al., 2023). It is also possible the MCM upregulation is reflecting an increase in DNA repair, which is associated with neurons undergoing synaptic strengthening (Madabhushi et al., 2015; Jovasevic et al., 2024). Indeed, overlapping the most significantly changed gene sets in cLTP and *Syngap^+/−^* CA1-TRAP populations reveals similar upregulation in DNA and chromatin regulatory processes (Fig. 2*F*,*G*; Extended Data Table 2-3).

Exaggerated, protein synthesis-independent mGluR-LTD has been seen in the *Syngap^+/−^* hippocampus (Barnes et al., 2015), which contrasts with the molecular similarities we observe between the *Syngap*^+/−^ translation profile and that of induced cLTP. Our examination of the *Syngap^+/−^* CA1 translatome does not reveal any obvious similarities to the mGluR-LTD stimulated translatome and instead shows a profile more aligned with LTP. Nonetheless, we cannot rule out the potential contribution of excessive protein synthesis to the *Syngap^+/−^* phenotype. An alternative explanation for this discrepancy may lie in altered AMPA receptor (AMPAR) dynamics in *Syngap*^+/−^ synapses, where the persistent synaptic strengthening and increase in AMPARs at the PSD in *Syngap^+/−^* CA1 neurons results in a greater loss of AMPARs from the postsynaptic membrane upon mGluR stimulation resulting in exaggerated LTD. Consequently, even though the molecular profile of *Syngap^+/−^* CA1 neurons aligns with cLTP, the structural and receptor composition changes in *Syngap^+/−^* synapses may facilitate an exaggerated mGluR-LTD response. In this context, elevated AMPAR availability could prime *Syngap*^+/−^ neurons for heightened synaptic weakening upon mGluR-LTD induction, resulting in an LTD response that is amplified despite the cLTP-like translation profile. Further experiments are necessary to tease apart this mechanism.

Along with the changes in DNA regulators, the transcripts convergently upregulated in *Syngap^+/−^* and cLTP-stimulated WT include regulators of synaptic function and cell adhesion (Fig. 2*E*). Further analyses revealed that both the *Syngap^+/−^* CA1 neurons and those stimulated for cLTP exhibit a significant increase in long (>2 kb) mRNAs in the translating fraction (Fig. 5*A*,*C*). This is opposite to a reduction in >2 kb transcripts we observe in *Fmr1^−/y^* CA1 pyramidal neurons and those stimulated for mGluR-LTD in WT (Fig. 5*B*,*D*). Importantly, this >2 kb population is enriched for regulators of synaptic function and cell adhesion (Fig. 5*E*,*F*), which is consistent with the inherent association of gene length and cellular function that has been described in the neuronal genome (Zylka et al., 2015; Seo et al., 2022). Previously, Seo et al. hypothesized that the reduced translation of long transcripts in *Fmr1^−/y^* neurons is proximal to increased ribosome abundance, which unequally impacts translation of mRNAs as a function of length (Seo et al., 2022). Others have suggested that the reduced translation of long mRNAs is due to impaired stability of long mRNAs in the absence of FMRP (Zalfa et al., 2007; Sawicka et al., 2019; Kurosaki et al., 2024). While we do not know the cause of the increased translation of long mRNAs in the *Syngap^+/−^* CA1-TRAP, there are significant changes in RNA binding proteins that may be involved in RNA stability including *Rbm38*, *Rbm47*, *RbmS3*, *Tent2*, and *Pabpc4*. It is also worth noting that DNA repair mechanisms are particularly relevant for longer genes, and the upregulation of MCM may selectively stabilize the transcription of these genes in *Syngap^+/−^* neurons (Stoeger et al., 2022; Soheili-Nezhad et al., 2024).

A closer look at the commonly upregulated >2 kb genes in cLTP and *Syngap^+/−^* hippocampal TRAP datasets reveal *Gigyf1*, *Tnks*, and *Setd2*—genes with known or putative roles in neurodevelopment and synaptic regulation. *Gigyf1* regulates IGF-1R/ERK signaling and has been implicated in ASD-relevant behaviors and neuronal subtype-specific functions (Giovannone et al., 2003; Chen et al., 2022; Ding et al., 2023). It also participates in translation-coupled mRNA decay through its interaction with 4EHP (Weber et al., 2020). *Tnks* has been shown to regulate proteasome activity via PI31 (Cho-Park and Steller, 2013) and also contributes to Wnt signaling, while *Setd2* plays dual roles in chromatin modification (H3K36me3) and cytoskeletal regulation (Koenning et al., 2021; Xu et al., 2021). In contrast, several >2 kb transcripts downregulated with LTD and basally in *Fmr1^−/y^* neurons—such as *Lrp1*, *Sez6l*, *Grik3*, and *Pcdhgc5*—are implicated in maintaining synaptic stability, trafficking of AMPA/NMDA receptors, or dendritic spine architecture (Liu et al., 2010; Nash et al., 2020; Pigoni et al., 2020; Su et al., 2024). These opposing regulatory patterns further support the notion that long mRNAs play a functional role in modulating synaptic plasticity state and its dysregulation in disease.

It is tempting to speculate that mechanisms that bias translation toward or away from long transcripts could be a type of gain control that allows specific plasticity states to be supported. These gene-level findings support our interpretation that the translational landscape in *Syngap^+/−^* reflects a constitutively LTP-biased state, while the *Fmr1^−/y^* profile resembles a chronic LTD-like state. However, further functional validation will be needed to confirm causal roles for these candidate genes in modulating plasticity phenotypes and disease-relevant behaviors.

Although our experiments do not distinguish between local/dendritic changes versus somatic changes, it is not unreasonable to suggest that such changes could be present in the local environment. Future experiments dissecting the local translating population during LTP or LTD stimulation would be particularly enlightening for spatial dynamics of this regulatory shift.

## Data Availability

All processed data is provided with manuscript as extended data files and figures. Transcriptomics sequencing raw data will be deposited in the Gene Expression Omnibus (GEO) upon publication. For analyses of cLTP, mGluR-LTD, and *Fmr1^−/y^* TRAP-seq, the following published datasets were used: GSE74985, GSE79790, GSE201239, and GSE101823.

## Synthesis

Reviewing Editor: Arianna Maffei, Stony Brook University Decisions are customarily a result of the Reviewing Editor and the peer reviewers coming together and discussing their recommendations until a consensus is reached. When revisions are invited, a fact-based synthesis statement explaining their decision and outlining what is needed to prepare a revision will be listed below. The following reviewer(s) agreed to reveal their identity: Ruchi Malik, Zoltan Simandi.

The manuscript was evaluated by 2 reviewers who agree on the significance of the study. There is agreement that the manuscript provides an important contribution to the field and that the study is overall well presented. The reviewers indicated the need to include more details about the analysis and specific information about the R procedures used. There are some concerns that the analysis of the mRNA many not fully reflect changes at the protein level. A thorough discussion about the findings in the context of extant literature and of the study limitations would strengthen the manuscript.

Reviewer #1

In this manuscript, the authors have systematically analyzed translating mRNAs of hippocampal CA1 neurons in two mouse models of autism: Syngap and Fmr1. The manuscript addresses a crucial question: How do alterations in protein synthesis in these mouse models affect plasticity phenotypes, Long-Term Potentiation (LTP) and Long-Term Depression (LTD)? The major findings suggest enrichment of translating mRNAs corresponding to persistent synaptic strengthening in neurons from Syngap mice. Interestingly, the authors observed that the basal translating mRNA population in Syngap mice mimics that induced by chemical LTP (but not mGluR-LTD). Similarly, the manuscript shows overlap between basal translating mRNAs in Fmr1 neurons and neurons that underwent LTD. Importantly, the authors report broadly opposite translatomes in 1) neurons from Syngap and Fmr1 mice and 2) CA1 neurons from wild-type mice after undergoing LTP and LTD protocols. This is a very interesting and well-designed study with important implications for understanding the mechanisms of aberrant plasticity in mouse models of autism.

The manuscript is well-written and the data are presented clearly. However, there are a few moderate issues that the authors need to address to further enhance the manuscript:

1) Correlation plots in figures 1G, 2B, 3B, 5C, 5D: The authors have used linear regression analysis to quantify the correlation between mRNA fold change and other related parameters from various experimental groups. However, given the scatter of the data and the reported correlation coefficients (r), the correlations appear to be very weak. In fact, there seems to be bimodal distribution of the data points along the axis in these plots, raising concerns about the appropriateness of linear regression analysis and its interpretation of these correlations. This issue should be addressed in the revised manuscript. Specifically, the authors should report the adjusted explained variance (R2) in their regression analysis. This metric quantifies the proportion of the variance of one variable (e.g., Syngap fold change) explained by the other variable (e.g., LTP fold change).

Additionally, I suggest that the authors explore fitting these data with non-linear models or Generalized Additive Models to better understand the relationship of different parameters investigated.

2) The cLTP dataset from Chen et al, 2017 reports LTP dependent change in mRNAs in both CA3 and CA1 neurons. Comparisons between the mRNA data from this mixed cell population dataset and CA1-only data from Syngap mice is less than a ideal analysis design because the CA3 and CA1 cell populations are molecularly distinct. The authors should acknowledge this limitation in the discussion section of the revised manuscript.

Relatedly, in the previously published LTP and LTD datasets used in this study, were the data collected from the entire dorsoventral hippocampus. The LTP/LTD properties and molecular profiles of CA1 neurons in dorsal and ventral parts of the hippocampus are known to be very different. Since the Syngap/Fmr1 dataset generated in this manuscript comes from the entire hippocampus, it will be important to ascertain whether the cLTP/LTD datasets also come from slices from both dorsal and ventral parts of the hippocampus.

3) On page 10 (lines 238-239), the authors mention "we removed transcripts that were also significantly changed in LTD dataset, resulting in a "LTP-specific" population". For clarity on the analysis parameters, could the authors provide more details/list of the transcripts that were excluded here?

4) The authors should mention the types of plots in the figure legends at several places in the manuscript (e.g. fig. 2C -- box plots with median and percentiles).

Reviewer #2

The authors of this study investigated the translational profiles of CA1 pyramidal neurons in Syngap+/- and Fmr1-/y mouse models, both relevant to neurodevelopmental disorders. Using TRAP-seq, the authors revealed that Syngap+/- neurons exhibit increased translation of long mRNAs (>2kb), mirroring changes observed during chemically induced LTP (cLTP) in wild-type (WT) neurons. Conversely, Fmr1-/y neurons showed decreased translation of long mRNAs, resembling changes during mGluR-LTD. Crucially, the study demonstrated that the Syngap+/- mutation primarily affects translational efficiency, rather than total mRNA abundance. This work highlights a distinct molecular signature for LTP and LTD, and suggests that Syngap+/- and Fmr1-/y models recapitulate LTP and LTD saturated states, respectively.

While the study has some important limitations, the authors acknowledge most of them in their discussion and overall, this work provides a strong foundation for further investigation into the molecular basis of synaptic plasticity and neurodevelopmental disorders.

The study presents several novel findings:

1) The identification of a distinct translational profile in Syngap+/- CA1 neurons, characterized by increased translation of long mRNAs and upregulation of DNA regulators, which is different from Fmr1-/y, is a significant contribution.

2) The discovery of opposite length-dependent shifts in mRNA translation (increased long mRNAs in LTP, decreased in LTD) is a novel aspect.

3) The study provides a clear molecular basis for the distinct synaptic plasticity phenotypes observed in these two models.

4) The study identifies specific candidate genes that may contribute to the occlusion of LTP or LTD in each model.

Major Criticisms:

1) TRAP-seq measures RNA abundance and ribosome association, not direct translation. The lack of direct protein level measurements weakens conclusions about protein synthesis. Direct protein level measurements are suggested to validate TRAP-seq findings and confirm protein changes.

2) Similarly, the study does not distinguish between somatic and dendritic translation, a critical factor in synaptic plasticity. Because local dendritic translation plays a critical role in synaptic responses, this represents an important gap. This could be addressed by visualizing and analyzing local translation using direct or indirect methods to determine protein or RNA levels.

3) Ideally, functional validation, such as CRISPR-Cas9 knockdown of specific genes and electrophysiological recordings to assess synaptic strength, would strengthen the correlation between mRNA changes and plasticity phenotypes. While acknowledging challenges to complete this in a timely manner, the reviewer recommends the authors to do a thorough review of existing literature for supporting data and extend their discussion.

4) The reporting of statistical methods lacks sufficient detail. Providing specific R package versions, as well as detailed information about data preprocessing and quality control, would significantly enhance the transparency and reproducibility of the study. For example, the description of DESeq2 analysis should be more detailed.

Minor criticisms:

1) Using mice aged P25-32 introduces a potential source of variability. While the use of littermates minimizes genetic variability, a tighter age range could have improved consistency.

2) The use of chemical agonists for LTP and LTD induction in hippocampal slices may not fully represent physiological synaptic activity.

## Author Response

Editor Comment:

Synthesis of Reviews:

Computational Neuroscience Model Code Accessibility Comments for Author (Required): N/A Synthesis Statement for Author (Required):

The manuscript was evaluated by 2 reviewers who agree on the significance of the study. There is agreement that the manuscript provides an important contribution to the field and that the study is overall well presented. The reviewers indicated the need to include more details about the analysis and specific information about the R procedures used. There are some concerns that the analysis of the mRNA many not fully reflect changes at the protein level. A thorough discussion about the findings in the context of extant literature and of the study limitations would strengthen the manuscript.

Author Response:

We thank the Editor for the supportive summary and for highlighting key areas for revision. We are also very grateful to the reviewers for carefully reviewing our manuscript and for their shared view of its significance and contribution to the field. In response to the points raised:

- We have expanded the Methods section to include detailed descriptions of our data analysis pipeline, including specific R package versions, analysis parameters, and quality control steps.

- We have added a more explicit discussion of the limitations of TRAP-seq, including the distinction between ribosome association and actual protein output, and acknowledged that complementary proteomic validation would further strengthen our conclusions.

- We have also expanded the Discussion to more thoroughly place our findings in the context of existing literature, including functional data from previous studies on key genes and mechanisms implicated in synaptic plasticity in Syngap+/- and Fmr1-/y models.

We appreciate the thoughtful and constructive feedback, and believe that the revisions have improved the clarity, rigor, and contextual depth of the manuscript. Following is a point-by-point response to their individual concerns. In the revised manuscript, we highlight changes in yellow in our manuscript text.

Reviewer #1 Reviewer Comment:

In this manuscript, the authors have systematically analyzed translating mRNAs of hippocampal CA1 neurons in two mouse models of autism: Syngap and Fmr1. The manuscript addresses a crucial question: How do alterations in protein synthesis in these mouse models affect plasticity phenotypes, Long-Term Potentiation (LTP) and Long-Term Depression (LTD)? The major findings suggest enrichment of translating mRNAs corresponding to persistent synaptic strengthening in neurons from Syngap mice. Interestingly, the authors observed that the basal translating mRNA population in Syngap mice mimics that induced by chemical LTP (but not mGluR-LTD). Similarly, the manuscript shows overlap between basal translating mRNAs in Fmr1 neurons and neurons that underwent LTD. Importantly, the authors report broadly opposite translatomes in 1) neurons from Syngap and Fmr1 mice and 2) CA1 neurons from wild-type mice after undergoing LTP and LTD protocols. This is a very interesting and well-designed study with important implications for understanding the mechanisms of aberrant plasticity in mouse models of autism.

The manuscript is well-written and the data are presented clearly. However, there are a few moderate issues that the authors need to address to further enhance the manuscript:

Author Response:

We sincerely thank the reviewer for their positive and encouraging feedback. We are especially grateful for the recognition of the conceptual framework, clarity of data presentation, and the novelty of our findings regarding the opposing translational landscapes in Syngap1 and Fmr1 mutant mouse models, and their resemblance to LTP and LTD states, respectively. We appreciate the reviewer's comments highlighting the relevance of this work to understand mechanisms of aberrant synaptic plasticity in autism models.

We have addressed the moderate issues raised below and believe these revisions further strengthen the manuscript.

Reviewer Comment:

1) Correlation plots in figures 1G, 2B, 3B, 5C, 5D: The authors have used linear regression analysis to quantify the correlation between mRNA fold change and other related parameters from various experimental groups. However, given the scatter of the data and the reported correlation coefficients (r), the correlations appear to be very weak. In fact, there seems to be bimodal distribution of the data points along the axis in these plots, raising concerns about the appropriateness of linear regression analysis and its interpretation of these correlations. This issue should be addressed in the revised manuscript. Specifically, the authors should report the adjusted explained variance (R2) in their regression analysis. This metric quantifies the proportion of the variance of one variable (e.g., Syngap fold change) explained by the other variable (e.g., LTP fold change).

Additionally, I suggest that the authors explore fitting these data with non-linear models or Generalized Additive Models to better understand the relationship of different parameters investigated.

Author Response:

We thank the reviewer for this thoughtful comment and agree that it is important to critically assess the strength and nature of the correlations reported. In response, we have now:

- Reported the adjusted R2 values for each linear regression analysis in Figures 1G, 2B, 3B, 5C, and 5D, and additionally 5A-B. which provide a more accurate measure of explained variance, especially given the different sample sizes across comparisons.

- Re-evaluated the data distributions in the mentioned plots. While the overall correlations remain modest (as reflected in the r and adjusted R² values), we agree with the reviewer that certain datasets display non-linear patterns and potential bimodal clustering. To address this, we performed exploratory analyses using Generalized Additive Models (GAMs) to evaluate non-linear trends in the data. In most comparisons, the GAM fits closely overlapped with the linear regression lines. However, in some cases, the GAMs provided improved fits (as reflected in deviance explained), suggesting heterogeneity in the regulation of a subset of transcripts.

These findings and revised plots are now included in the updated manuscript (main Figures 1, 2, 3, 5 and extended Figure1-3, Figure2-1, Figure3-1, Figure5-1), along with text description in the Results section. We appreciate the reviewer's suggestion, which has strengthened the rigor and interpretability of our correlation analyses.

Reviewer Comment:

2) The cLTP dataset from Chen et al, 2017 reports LTP dependent change in mRNAs in both CA3 and CA1 neurons. Comparisons between the mRNA data from this mixed cell population dataset and CA1-only data from Syngap mice is less than a ideal analysis design because the CA3 and CA1 cell populations are molecularly distinct. The authors should acknowledge this limitation in the discussion section of the revised manuscript.

Relatedly, in the previously published LTP and LTD datasets used in this study, were the data collected from the entire dorsoventral hippocampus. The LTP/LTD properties and molecular profiles of CA1 neurons in dorsal and ventral parts of the hippocampus are known to be very different. Since the Syngap/Fmr1 dataset generated in this manuscript comes from the entire hippocampus, it will be important to ascertain whether the cLTP/LTD datasets also come from slices from both dorsal and ventral parts of the hippocampus.

Author Response:

We appreciate the reviewer's thoughtful comments regarding the anatomical specificity of the datasets used in our comparative analyses. We agree that CA3 and CA1 neurons are molecularly distinct, and that this presents a limitation when directly comparing the mixed CA3/CA1 cLTP dataset (Chen et al., 2017) to our CA1-enriched Syngap dataset. In response, we have now explicitly acknowledged this limitation in the revised Discussion and emphasized the value of future studies employing spatially restricted or cell-type-specific approaches (e.g., CA1-specific TRAP or RiboTag following cLTP stimulation) to enable more precise comparisons.

While the cLTP dataset includes transcripts from both CA3 and CA1 regions, the methods describe the preparation of "400 µm thick CA3/CA1 mini-slices" with dentate gyrus microdissection. Based on this, along with the use of transverse slicing, interface-type recovery, and the common focus of cLTP paradigms on CA1, it is likely that a substantial portion of the signal in this dataset arises from dorsal CA1. Similarly, the LTD dataset (Seo et al., 2022) used comparable preparation strategies that are also biased toward dorsal hippocampus.

In contrast, our Syngap+/- and Fmr1-/y datasets were generated from tissue spanning the entire dorsoventral axis of the hippocampus. We now acknowledge this anatomical difference in the Discussion as a potential limitation for region-specific comparisons. However, dorsal CA1 is known to exhibit stronger plasticity responses and higher translational activity compared to ventral regions. As such, we expect that dorsal-derived signals contribute substantially to our data, supporting the validity of comparisons with the cLTP and LTD datasets. Moreover, our analyses focus on broad, gene-level patterns of translational regulation - such as length-dependent changes and gene set enrichment - that remain interpretable despite these anatomical differences.

Reviewer Comment:

3) On page 10 (lines 238-239), the authors mention "we removed transcripts that were also significantly changed in LTD dataset, resulting in a "LTP-specific" population". For clarity on the analysis parameters, could the authors provide more details/list of the transcripts that were excluded here? Author Response:

We thank the reviewer for pointing this out and agree that providing more detail will enhance clarity and reproducibility. In the revised manuscript, we have now added the exact criteria used to define "significantly changed" transcripts in the LTD and LTP datasets: adjusted p-value (padj) < 0.1 regardless of the direction of fold change. Any transcript that was significantly changed in both the LTP and LTD datasets was excluded from the LTP-induced gene list, resulting in a refined "LTP-specific" population. The same approach was applied in reverse to define the "LTD-specific" population.

To further improve transparency, we have now included the full list of transcripts excluded from the LTP list due to significant changes in the LTD dataset as extended Table 2-1.

Reviewer Comment:

4) The authors should mention the types of plots in the figure legends at several places in the manuscript (e.g. fig. 2C -- box plots with median and percentiles).

Author Response:

We thank the reviewer for this helpful suggestion. We have now revised the figure legends throughout the manuscript to clearly specify the type of plots used (box plots, vs scatter plots), including whether the plots display median, interquartile range, or full data distribution where applicable. Specifically, the legend for Figure 2C as well as 3C and extended figures has been updated to indicate that it shows a box plot with the median and 25th/75th percentiles. Similar clarifications have been made in all relevant figure legends to improve clarity and interpretability.

Reviewer #2 The authors of this study investigated the translational profiles of CA1 pyramidal neurons in Syngap+/- and Fmr1-/y mouse models, both relevant to neurodevelopmental disorders. Using TRAP-seq, the authors revealed that Syngap+/- neurons exhibit increased translation of long mRNAs (>2kb), mirroring changes observed during chemically induced LTP (cLTP) in wild- type (WT) neurons. Conversely, Fmr1-/y neurons showed decreased translation of long mRNAs, resembling changes during mGluR-LTD. Crucially, the study demonstrated that the Syngap+/- mutation primarily affects translational efficiency, rather than total mRNA abundance. This work highlights a distinct molecular signature for LTP and LTD, and suggests that Syngap+/- and Fmr1-/y models recapitulate LTP and LTD saturated states, respectively.

While the study has some important limitations, the authors acknowledge most of them in their discussion and overall, this work provides a strong foundation for further investigation into the molecular basis of synaptic plasticity and neurodevelopmental disorders.

The study presents several novel findings:

1) The identification of a distinct translational profile in Syngap+/- CA1 neurons, characterized by increased translation of long mRNAs and upregulation of DNA regulators, which is different from Fmr1-/y, is a significant contribution.

2) The discovery of opposite length-dependent shifts in mRNA translation (increased long mRNAs in LTP, decreased in LTD) is a novel aspect.

3) The study provides a clear molecular basis for the distinct synaptic plasticity phenotypes observed in these two models.

4) The study identifies specific candidate genes that may contribute to the occlusion of LTP or LTD in each model.

Author Response:

We sincerely thank the reviewer for their thoughtful summary and appreciation of our work. We are especially grateful for the recognition of the novel aspects of our study, including the identification of opposing length-dependent translational shifts in Syngap+/- and Fmr1-/y neurons, the distinct molecular signatures reflective of LTP- and LTD-like states, and the emphasis on translational efficiency rather than mRNA abundance. We are encouraged that the reviewer views this work as a strong foundation for further investigation into the molecular basis of synaptic plasticity and neurodevelopmental disorders.

Major Criticisms:

Reviewer Comment:

1) TRAP-seq measures RNA abundance and ribosome association, not direct translation. The lack of direct protein level measurements weakens conclusions about protein synthesis. Direct protein level measurements are suggested to validate TRAP-seq findings and confirm protein changes.

Author Response:

We appreciate the reviewer's important point regarding the distinction between ribosome association and direct protein output. While it is true that TRAP-seq measures ribosome-bound mRNAs rather than protein levels per se, it remains a widely accepted and sensitive method for capturing changes in translational efficiency and potential protein synthesis at a cell-type-specific resolution, which is particularly valuable in neuronal populations that are otherwise difficult to isolate.

Given the technical challenges and limitations associated with acquiring cell-type-specific proteomic data from CA1 pyramidal neurons in vivo, we focused this study on a robust transcriptome-wide analysis of ribosome engagement using TRAP. Our primary conclusions center on relative shifts in translational regulation and are supported by strong internal consistency with known physiological and molecular phenotypes in Syngap+/- and Fmr1-/y models. While we agree that direct protein measurements would further strengthen the study, we believe that the TRAP-based findings provide meaningful insight into the translational landscape in these models.

We had already noted the limitation of TRAP-seq in Discussion section, but we have now expanded this to explicitly acknowledge that our approach reflect a potential change at protein level and future studies using complementary proteomic approaches could help further validate and extend these findings.

Reviewer Comment:

2) Similarly, the study does not distinguish between somatic and dendritic translation, a critical factor in synaptic plasticity. Because local dendritic translation plays a critical role in synaptic responses, this represents an important gap. This could be addressed by visualizing and analyzing local translation using direct or indirect methods to determine protein or RNA levels.

Author Response:

We thank the reviewer for highlighting the importance of compartment-specific translation, particularly the role of local dendritic protein synthesis in synaptic plasticity. While our TRAP-seq approach does not spatially resolve somatic and dendritic compartments, we were curious and had already performed an exploratory analysis using a previously published annotation of transcripts (Glock, Biever et al, PNAS 2021) enriched in either neuropil ("npl-translation-up") or soma ("smt-translation-up") compartments. In the LTP dataset, neuropil-enriched transcripts were more strongly upregulated than somatic ones, consistent with activity-dependent local translation. However, when we examined these same categories in the Syngap+/- translatome, the pattern was less distinct and did not support a strong localization bias.

As such, we chose not to include these findings in the manuscript to avoid overinterpretation. We had already mentioned this limitation explicitly in the Discussion and emphasized that "Future experiments dissecting the local translating population during LTP or LTD stimulation would be particularly enlightening for answering this question".

Reviewer Comment:

3) Ideally, functional validation, such as CRISPR-Cas9 knockdown of specific genes and electrophysiological recordings to assess synaptic strength, would strengthen the correlation between mRNA changes and plasticity phenotypes. While acknowledging challenges to complete this in a timely manner, the reviewer recommends the authors to do a thorough review of existing literature for supporting data and extend their discussion.

Author Response:

We thank the reviewer for this thoughtful and constructive suggestion. We fully agree that functional validation, such as gene-specific perturbation and electrophysiological assessment, would be a powerful way to link the observed translational changes to synaptic function. While these experiments are beyond the current scope of this study, we appreciate the importance of reinforcing our findings with supporting evidence.

In response, we have now performed a more thorough literature review of key genes identified in our analysis, particularly those with potential roles in synaptic plasticity, LTP/LTD modulation, or known phenotypes in Syngap+/- and Fmr1-/y models. We have expanded the Discussion to include relevant studies that provide indirect or direct evidence supporting their functional roles, helping to contextualize the molecular findings within known physiological frameworks.

We thank the reviewer for encouraging us to strengthen this aspect of the manuscript, and we believe these additions further solidify the translational relevance of our findings.

Reviewer Comment:

4) The reporting of statistical methods lacks sufficient detail. Providing specific R package versions, as well as detailed information about data preprocessing and quality control, would significantly enhance the transparency and reproducibility of the study. For example, the description of DESeq2 analysis should be more detailed.

Author Response:

We thank the reviewer for this important suggestion regarding the reporting of statistical methods. In the revised manuscript, we have carefully reviewed and expanded the Methods section to enhance transparency and reproducibility.

Specifically, we now provide additional detail regarding our DESeq2 analysis in "RNA-Seq library preparation and analysis" section, including the design formula (~ Pair + Condition), the filtering of lowly expressed genes (requiring at least 10 normalized counts in a minimum of 3 samples), and the use of the "normal" log2 fold change shrinkage estimator for visualization and ranking.

Furthermore, we clarify that all datasets (Syngap+/−, Fmr1-/y, cLTP, and mGluR-LTD) were analyzed separately using paired experimental designs, and that normalization and statistical testing were performed using DESeq2's default settings unless otherwise specified.

Each section of the Methods already includes the exact software tools and version numbers used for RNA-seq processing, quality control, and analysis (e.g., Cutadapt v2.6, FastQC v0.11.9, MultiQC v1.10.1, STAR v2.7.10b, featureCounts v2.0.5, and DESeq2 v1.40.1). In addition, we have now mentioned specific parameters used for adapter removal and alignment using STAR. We believe these revisions now address the reviewer's request and strengthen the reproducibility of our computational workflow.

Minor criticisms:

Reviewer Comment:

1) Using mice aged P25-32 introduces a potential source of variability. While the use of littermates minimizes genetic variability, a tighter age range could have improved consistency.

Author Response:

We thank the reviewer for this observation. We agree that developmental stage can influence transcriptional and translational profiles, particularly during early postnatal periods. In this study, we selected mice aged P25-32 to capture a window of postnatal maturation where synaptic plasticity mechanisms are robustly engaged but before significant aging-related changes emerge. Importantly, all comparisons were made between genotype-matched littermates within the same age range, and animals were age- matched within each experimental group to minimize variability.

We have now added a note in the Methods section acknowledging this potential source of variation and justifying our rationale for selecting this age range. We also note that the overall patterns and effect sizes observed across replicates suggest that age-related variability was not a major confounding factor in our dataset.

Reviewer Comment:

2) The use of chemical agonists for LTP and LTD induction in hippocampal slices may not fully represent physiological synaptic activity.

Author Response:

We thank the reviewer for raising this important point. We agree that chemical induction of LTP and LTD may not fully replicate the physiological patterns of synaptic activity that occur in vivo. As already noted in the Discussion, we had acknowledged this as a limitation of the study. While chemical stimulation paradigms (forskolin or DHPG) can have broader cellular effects beyond synaptic plasticity, they are widely used in the field to elicit robust and reproducible potentiation or depression, particularly in slice preparations.

Importantly, we observed a consistent upregulation of immediate early genes (IEGs) in both LTP and LTD datasets (Fig. 4C), suggesting that overall neuronal activation occurred in both conditions. This supports the idea that the opposing transcriptomic changes observed are not solely due to differential activity levels. We have retained this clarification in the Discussion and believe it appropriately frames the interpretive boundaries of these datasets.

## References

[B1] Araki Y, et al. (2024) SynGAP regulates synaptic plasticity and cognition independently of its catalytic activity. Science 383:eadk1291. 10.1126/science.adk1291 38422154 PMC11188940

[B2] Araki Y, Hong I, Gamache TR, Ju S, Collado-Torres L, Shin JH, Huganir RL (2020) SynGAP isoforms differentially regulate synaptic plasticity and dendritic development. Elife 9:e56273. 10.7554/eLife.56273 32579114 PMC7314543

[B3] Araki Y, Zeng M, Zhang M, Huganir RL (2015) Rapid dispersion of SynGAP from synaptic spines triggers AMPA receptor insertion and spine enlargement during LTP. Neuron 85:173–189. 10.1016/j.neuron.2014.12.023 25569349 PMC4428669

[B4] Aryal S, Longo F, Klann E (2021) Genetic removal of p70 S6K1 corrects coding sequence length-dependent alterations in mRNA translation in fragile X syndrome mice. Proc Natl Acad Sci U S A 118:e2001681118. 10.1073/pnas.2001681118 33906942 PMC8106352

[B5] Auerbach BD, Bear MF (2010) Loss of the fragile X mental retardation protein decouples metabotropic glutamate receptor dependent priming of long-term potentiation from protein synthesis. J Neurophysiol 104:1047–1051. 10.1152/jn.00449.2010 20554840 PMC2934918

[B6] Barnes SA, et al. (2015) Convergence of hippocampal pathophysiology in Syngap+/- and Fmr1-/y mice. J Neurosci 35:15073–15081. 10.1523/JNEUROSCI.1087-15.2015 26558778 PMC4642239

[B7] Bear MF, Huber KM, Warren ST (2004) The mGluR theory of fragile X mental retardation. Trends Neurosci 27:370–377. 10.1016/j.tins.2004.04.009 15219735

[B8] Birtele M, et al. (2023) Non-synaptic function of the autism spectrum disorder-associated gene SYNGAP1 in cortical neurogenesis. Nat Neurosci 26:2090–2103. 10.1038/s41593-023-01477-3 37946050 PMC11349286

[B9] Chen G, et al. (2022) GIGYF1 disruption associates with autism and impaired IGF-1R signaling. J Clin Invest 132:e159806. 10.1172/JCI159806 35917186 PMC9525121

[B10] Chen PB, Kawaguchi R, Blum C, Achiro JM, Coppola G, Dell O, Martin TJ, C K (2017) Mapping gene expression in excitatory neurons during hippocampal late-phase long-term potentiation. Front Mol Neurosci 10:39. 10.3389/fnmol.2017.00039 28275336 PMC5319997

[B11] Chen EY, Tan CM, Kou Y, Duan Q, Wang Z, Meirelles GV, Clark NR, Ma'ayan A (2013) Enrichr: interactive and collaborative HTML5 gene list enrichment analysis tool. BMC Bioinformatics 14:128. 10.1186/1471-2105-14-128 23586463 PMC3637064

[B12] Cho-Park PF, Steller H (2013) Proteasome regulation by ADP-ribosylation. Cell 153:614–627. 10.1016/j.cell.2013.03.040 23622245 PMC3676968

[B13] Cole AJ, Saffen DW, Baraban JM, Worley PF (1989) Rapid increase of an immediate early gene messenger RNA in hippocampal neurons by synaptic NMDA receptor activation. Nature 340:474–476. 10.1038/340474a0 2547165

[B14] Davis HP, Squire LR (1984) Protein synthesis and memory: a review. Psychol Bull 96:518–559. 10.1037/0033-2909.96.3.518 6096908

[B15] Ding Z, et al. (2023) Genetic ablation of GIGYF1, associated with autism, causes behavioral and neurodevelopmental defects in zebrafish and mice. Biol Psychiatry 94:769–779. 10.1016/j.biopsych.2023.02.993 36924980 PMC10502190

[B16] Dolen G, Osterweil E, Rao BS, Smith GB, Auerbach BD, Chattarji S, Bear MF (2007) Correction of fragile X syndrome in mice. Neuron 56:955–962. 10.1016/j.neuron.2007.12.001 18093519 PMC2199268

[B17] Gabel HW, Kinde B, Stroud H, Gilbert CS, Harmin DA, Kastan NR, Hemberg M, Ebert DH, Greenberg ME (2015) Disruption of DNA-methylation-dependent long gene repression in Rett syndrome. Nature 522:89–93. 10.1038/nature14319 25762136 PMC4480648

[B18] Giovannone B, Lee E, Laviola L, Giorgino F, Cleveland KA, Smith RJ (2003) Two novel proteins that are linked to insulin-like growth factor (IGF-I) receptors by the Grb10 adapter and modulate IGF-I signaling. J Biol Chem 278:31564–31573. 10.1074/jbc.M211572200 12771153

[B19] Greenberg ME, Ziff EB, Greene LA (1986) Stimulation of neuronal acetylcholine receptors induces rapid gene transcription. Science 234:80–83. 10.1126/science.3749894 3749894

[B20] Greenblatt EJ, Spradling AC (2018) Fragile X mental retardation 1 gene enhances the translation of large autism-related proteins. Science 361:709–712. 10.1126/science.aas9963 30115809 PMC6905618

[B21] Heiman M, et al. (2008) A translational profiling approach for the molecular characterization of CNS cell types. Cell 135:738–748. 10.1016/j.cell.2008.10.028 19013281 PMC2696821

[B22] Holt CE, Schuman EM (2013) The central dogma decentralized: new perspectives on RNA function and local translation in neurons. Neuron 80:648–657. 10.1016/j.neuron.2013.10.036 24183017 PMC3820025

[B23] Hou L, Antion MD, Hu D, Spencer CM, Paylor R, Klann E (2006) Dynamic translational and proteasomal regulation of fragile X mental retardation protein controls mGluR-dependent long-term depression. Neuron 51:441–454. 10.1016/j.neuron.2006.07.005 16908410

[B24] Huber KM, Gallagher SM, Warren ST, Bear MF (2002) Altered synaptic plasticity in a mouse model of fragile X mental retardation. Proc Natl Acad Sci U S A 99:7746–7750. 10.1073/pnas.122205699 12032354 PMC124340

[B25] Jovasevic V, et al. (2024) Formation of memory assemblies through the DNA-sensing TLR9 pathway. Nature 628:145–153. 10.1038/s41586-024-07220-7 38538785 PMC10990941

[B26] Kelleher RJ 3rd, Bear MF (2008) The autistic neuron: troubled translation? Cell 135:401–406. 10.1016/j.cell.2008.10.017 18984149

[B27] Kidd SA, Lachiewicz A, Barbouth D, Blitz RK, Delahunty C, McBrien D, Visootsak J, Berry-Kravis E (2014) Fragile X syndrome: a review of associated medical problems. Pediatrics 134:995–1005. 10.1542/peds.2013-4301 25287458

[B28] Kim JH, Liao D, Lau LF, Huganir RL (1998) SynGAP: a synaptic RasGAP that associates with the PSD-95/SAP90 protein family. Neuron 20:683–691. 10.1016/S0896-6273(00)81008-9 9581761

[B29] Koenning M, et al. (2021) Neuronal SETD2 activity links microtubule methylation to an anxiety-like phenotype in mice. Brain 144:2527–2540. 10.1093/brain/awab200 34014281 PMC8418347

[B30] Komiyama NH, et al. (2002) SynGAP regulates ERK/MAPK signaling, synaptic plasticity, and learning in the complex with postsynaptic density 95 and NMDA receptor. J Neurosci 22:9721–9732. 10.1523/JNEUROSCI.22-22-09721.2002 12427827 PMC6757832

[B31] Kurosaki T, Rambout X, Maquat LE (2024) FMRP-mediated spatial regulation of physiologic NMD targets in neuronal cells. Genome Biol 25:31. 10.1186/s13059-023-03146-x 38263082 PMC10804635

[B32] Liao Y, Smyth GK, Shi W (2014) Featurecounts: an efficient general purpose program for assigning sequence reads to genomic features. Bioinformatics 30:923–930. 10.1093/bioinformatics/btt656 24227677

[B33] Liu Q, Trotter J, Zhang J, Peters MM, Cheng H, Bao J, Han X, Weeber EJ, Bu G (2010) Neuronal LRP1 knockout in adult mice leads to impaired brain lipid metabolism and progressive, age-dependent synapse loss and neurodegeneration. J Neurosci 30:17068–17078. 10.1523/JNEUROSCI.4067-10.2010 21159977 PMC3146802

[B34] Llamosas N, et al. (2020) SYNGAP1 controls the maturation of dendrites, synaptic function, and network activity in developing human neurons. J Neurosci 40:7980–7994. 10.1523/JNEUROSCI.1367-20.2020 32887745 PMC7548701

[B35] Love MI, Huber W, Anders S (2014) Moderated estimation of fold change and dispersion for RNA-seq data with DESeq2. Genome Biol 15:1–21. 10.1186/gb-2014-15-1-r1 25516281 PMC4302049

[B36] Madabhushi R, et al. (2015) Activity-induced DNA breaks govern the expression of neuronal early-response genes. Cell 161:1592–1605. 10.1016/j.cell.2015.05.032 26052046 PMC4886855

[B37] Martin M (2011) Cutadapt removes adapter sequences from high-throughput sequencing reads. J Comput Biol 17:10–12. 10.14806/ej.17.1.200

[B38] McCoy MJ, Paul AJ, Victor MB, Richner M, Gabel HW, Gong H, Yoo AS, Ahn TH (2018) LONGO: an R package for interactive gene length dependent analysis for neuronal identity. Bioinformatics 34:i422–i428. 10.1093/bioinformatics/bty243 29950021 PMC6022641

[B39] Mei L, Cook JG (2021) Efficiency and equity in origin licensing to ensure complete DNA replication. Biochem Soc Trans 49:2133–2141. 10.1042/BST20210161 34545932 PMC9082567

[B40] Mignot C, et al. (2016) Genetic and neurodevelopmental spectrum of SYNGAP1-associated intellectual disability and epilepsy. J Med Genet 53:511–522. 10.1136/jmedgenet-2015-103451 26989088

[B41] Nash A, Aumann TD, Pigoni M, Lichtenthaler SF, Takeshima H, Munro KM, Gunnersen JM (2020) Lack of Sez6 family proteins impairs motor functions, short-term memory, and cognitive flexibility and alters dendritic spine properties. Cereb Cortex 30:2167–2184. 10.1093/cercor/bhz230 31711114

[B42] Nosyreva ED, Huber KM (2006) Metabotropic receptor-dependent long-term depression persists in the absence of protein synthesis in the mouse model of fragile X syndrome. J Neurophysiol 95:3291–3295. 10.1152/jn.01316.2005 16452252

[B43] Osterweil EK, Krueger DD, Reinhold K, Bear MF (2010) Hypersensitivity to mGluR5 and ERK1/2 leads to excessive protein synthesis in the hippocampus of a mouse model of fragile X syndrome. J Neurosci 30:15616–15627. 10.1523/JNEUROSCI.3888-10.2010 21084617 PMC3400430

[B44] Pigoni M, et al. (2020) Seizure protein 6 controls glycosylation and trafficking of kainate receptor subunits GluK2 and GluK3. EMBO J 39:e103457. 10.15252/embj.2019103457 32567721 PMC7396870

[B45] Qin M, Kang J, Burlin TV, Jiang C, Smith CB (2005) Postadolescent changes in regional cerebral protein synthesis: an in vivo study in the FMR1 null mouse. J Neurosci 25:5087–5095. 10.1523/JNEUROSCI.0093-05.2005 15901791 PMC6724856

[B46] Rumbaugh G, Adams JP, Kim JH, Huganir RL (2006) SynGAP regulates synaptic strength and mitogen-activated protein kinases in cultured neurons. Proc Natl Acad Sci U S A 103:4344–4351. 10.1073/pnas.0600084103 16537406 PMC1450173

[B47] Sawicka K, Hale CR, Park CY, Fak JJ, Gresack JE, Van Driesche SJ, Kang JJ, Darnell JC, Darnell RB (2019) FMRP has a cell-type-specific role in CA1 pyramidal neurons to regulate autism-related transcripts and circadian memory. Elife 8:e46919. 10.7554/eLife.46919 31860442 PMC6924960

[B48] Sedlackova H, Rask MB, Gupta R, Choudhary C, Somyajit K, Lukas J (2020) Equilibrium between nascent and parental MCM proteins protects replicating genomes. Nature 587:297–302. 10.1038/s41586-020-2842-3 33087936

[B49] Seo SS, et al. (2022) Excess ribosomal protein production unbalances translation in a model of fragile X syndrome. Nat Commun 13:3236. 10.1038/s41467-022-30979-0 35688821 PMC9187743

[B50] Soheili-Nezhad S, Ibanez-Sole O, Izeta A, Hoeijmakers JHJ, Stoeger T (2024) Time is ticking faster for long genes in aging. Trends Genet 40:299–312. 10.1016/j.tig.2024.01.009 38519330 PMC11003850

[B51] Stoeger T, et al. (2022) Aging is associated with a systemic length-associated transcriptome imbalance. Nat Aging 2:1191–1206. 10.1038/s43587-022-00317-6 37118543 PMC10154227

[B52] Su M, Xuan E, Sun X, Pan G, Li D, Zheng H, Zhang YW, Li Y (2024) Synaptic adhesion molecule protocadherin-gammaC5 mediates beta-amyloid-induced neuronal hyperactivity and cognitive deficits in Alzheimer's disease. J Neurochem 168:1060–1079. 10.1111/jnc.16066 38308496

[B53] Thomson SR, et al. (2017) Cell-type-specific translation profiling reveals a novel strategy for treating fragile X syndrome. Neuron 95:550–563.e5. 10.1016/j.neuron.2017.07.013 28772121 PMC5548955

[B54] Wang CC, Held RG, Hall BJ (2013) SynGAP regulates protein synthesis and homeostatic synaptic plasticity in developing cortical networks. PLoS One 8:e83941. 10.1371/journal.pone.0083941 24391850 PMC3877118

[B55] Weber R, Chung MY, Keskeny C, Zinnall U, Landthaler M, Valkov E, Izaurralde E, Igreja C (2020) 4EHP and GIGYF1/2 mediate translation-coupled messenger RNA decay. Cell Rep 33:108262. 10.1016/j.celrep.2020.108262 33053355 PMC8984682

[B56] Xu L, et al. (2021) Abnormal neocortex arealization and Sotos-like syndrome-associated behavior in Setd2 mutant mice. Sci Adv 7:eaba1180. 10.1126/sciadv.aba1180 33523829 PMC7775761

[B57] Yadav AK, Polasek-Sedlackova H (2024) Quantity and quality of minichromosome maintenance protein complexes couple replication licensing to genome integrity. Commun Biol 7:167. 10.1038/s42003-024-05855-w 38336851 PMC10858283

[B58] Yu G, Wang LG, Han Y, He QY (2012) Clusterprofiler: an R package for comparing biological themes among gene clusters. OMICS 16:284–287. 10.1089/omi.2011.0118 22455463 PMC3339379

[B59] Zalfa F, et al. (2007) A new function for the fragile X mental retardation protein in regulation of PSD-95 mRNA stability. Nat Neurosci 10:578–587. 10.1038/nn1893 17417632 PMC2804293

[B60] Zylka MJ, Simon JM, Philpot BD (2015) Gene length matters in neurons. Neuron 86:353–355. 10.1016/j.neuron.2015.03.059 25905808 PMC4584405

