# Supplementary figures and images for: Syngap+/− CA1 Pyramidal Neurons Exhibit Upregulated Translation of Long MRNAs Associated with LTP
Source: eNeuro. 2025 May 6;12(5):ENEURO.0086-25.2025. doi: 10.1523/ENEURO.0086-25.2025 (PMC12091090; doi:10.1523/ENEURO.0086-25.2025)

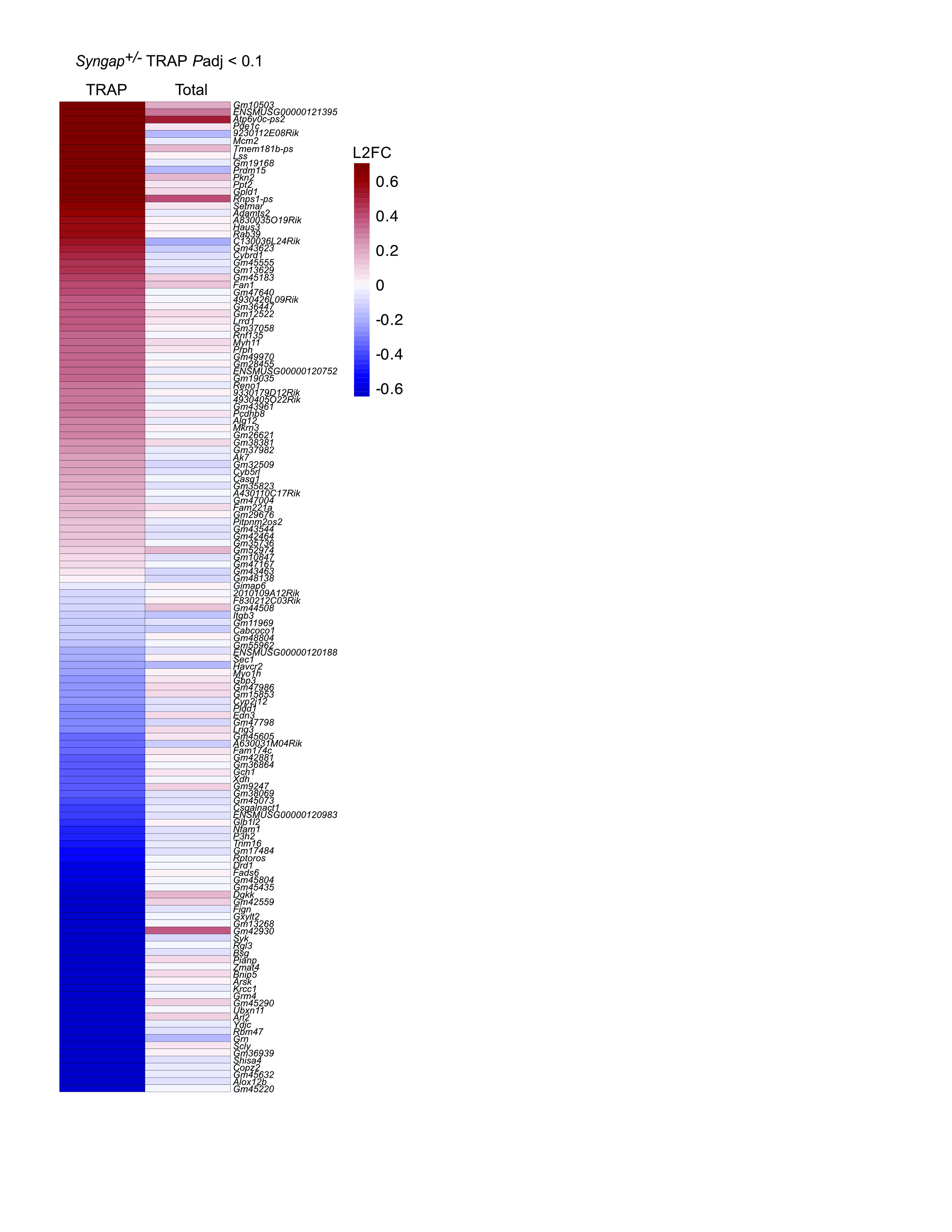

Supplement: Figure 1-1 — Significantly altered transcripts in Syngap+/- CA1-TRAP are not changed in total transcriptome. A heatmap of log2foldchanges (L2FC) shows that significant transcripts altered in Syngap+/- translatome are not changed similarly in the total transcriptome. Download Figure 1-1, TIF file. [file eneuro-12-ENEURO.0086-25.2025-s003.tif]

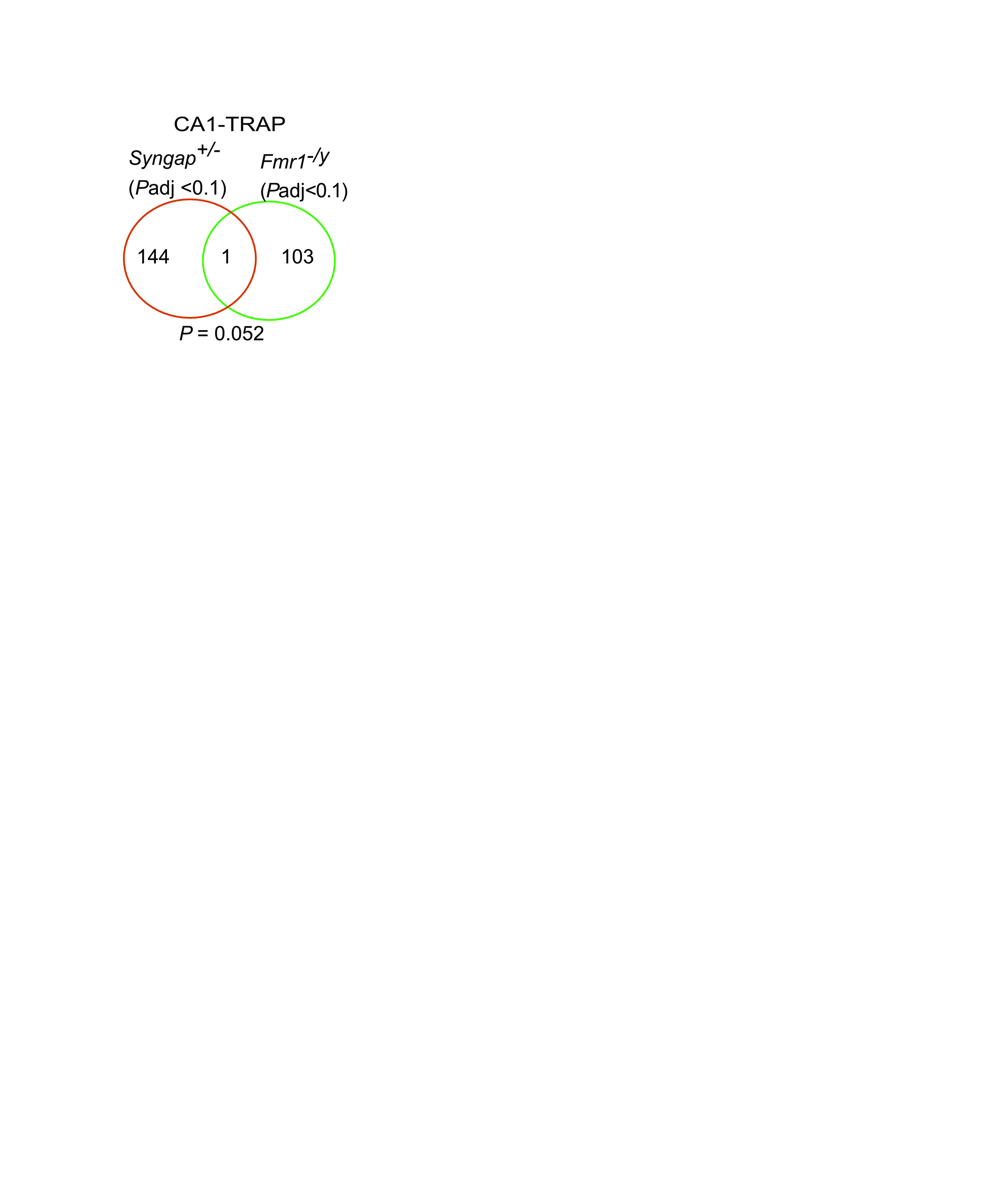

Supplement: Figure 1-2 — Mistranslation of distinct transcripts in Syngap+/ and Fmr1-/y. Quantification of transcripts shows 145 significant transcripts (Padj < 0.1) are differentially translating in Syngap+/- and only 1 of those overlap with the significant translatome in Fmr1-/y P = 0.052). Download Figure 1-2, TIF file. [file eneuro-12-ENEURO.0086-25.2025-s006.tif]

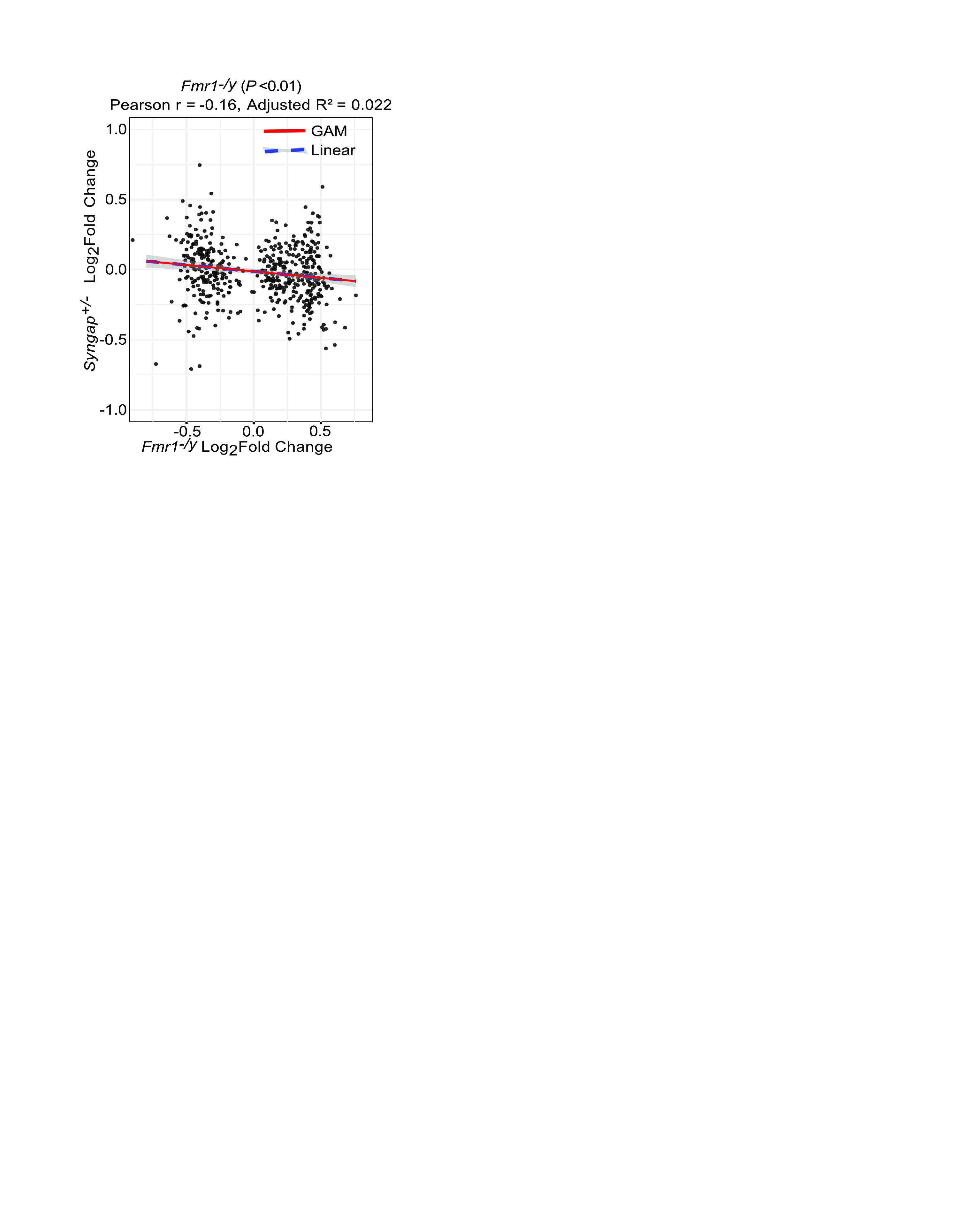

Supplement: Figure 1-3 — Non-linear modeling of the correlation between Syngap+/- and Fmr1-/y CA1-TRAP Log2 fold changes. This plot shows the relationship between transcripts significantly changed in the Fmr1-/y dataset (P < 0.01) and their corresponding fold changes in the Syngap+/- dataset. Both linear regression (blue dashed line) and Generalized Additive Model (GAM, red curve) fits are shown, with substantial overlap indicating that the relationship is largely captured by a linear model. Download Figure 1-3, TIF file. [file eneuro-12-ENEURO.0086-25.2025-s004.tif]

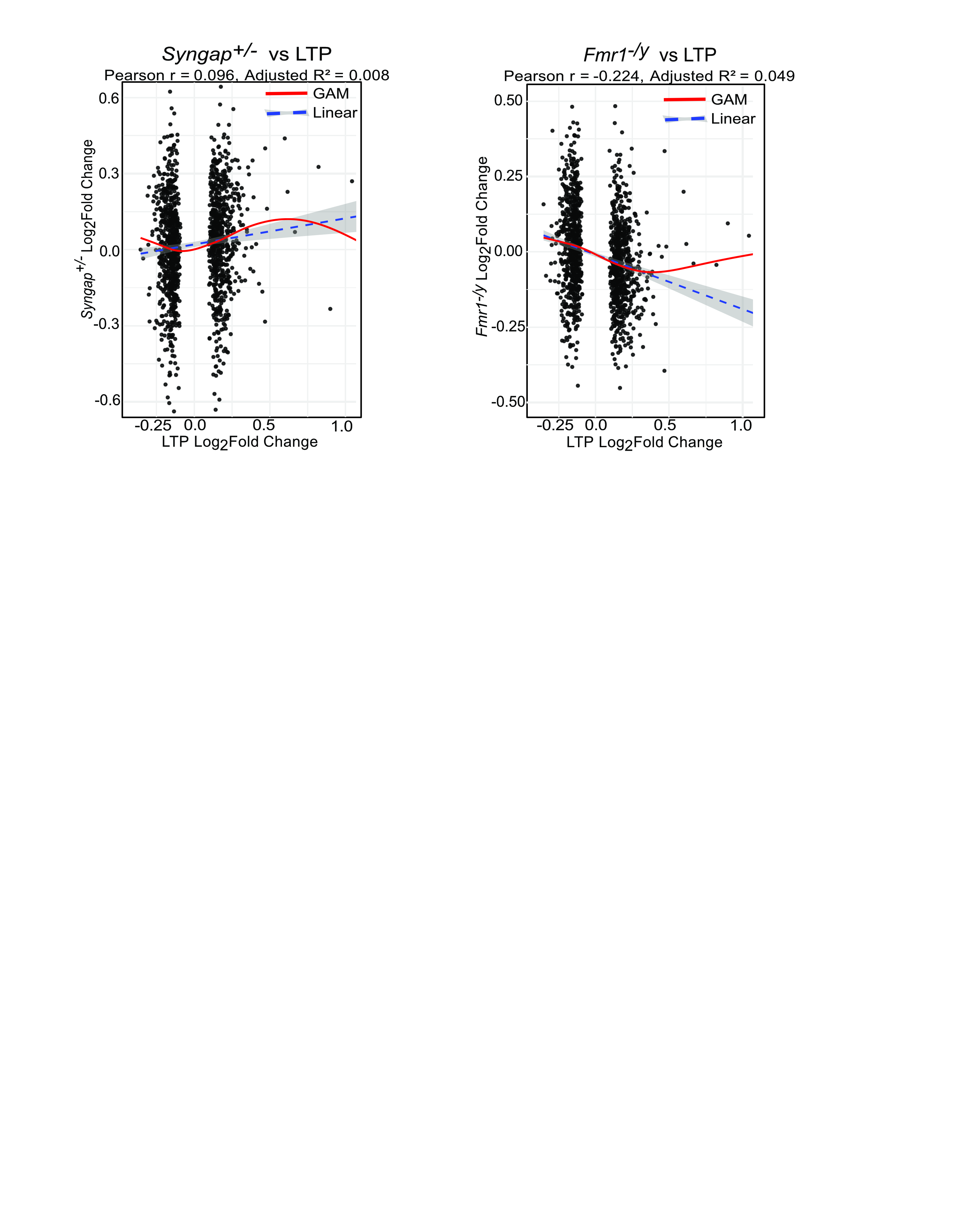

Supplement: Figure 2-1 — Non-linear modeling of the relationship between LTP-induced transcript changes and CA1-TRAP profiles in Syngap+/- and Fmr1-/y mice. Scatterplots show the Log2 fold changes of transcripts significantly altered in the LTP dataset (P < 0.01) plotted against their corresponding fold changes in Syngap+/- (left) and Fmr1-/y (right) CA1-TRAP datasets. Linear regression fits (blue dashed lines) and Generalized Additive Model (GAM) fits (red curves) are overlaid. In the Fmr1-/y comparison, a substantial overlap between the GAM and linear fits, indicates a predominantly linear inverse relationship. In contrast, the Syngap+/- comparison showed the deviation of the GAM curve from the linear trend suggests a non-uniform relationship, consistent with heterogeneous upregulation of LTP-induced genes. Download Figure 2-1, TIF file. [file eneuro-12-ENEURO.0086-25.2025-s008.tif]

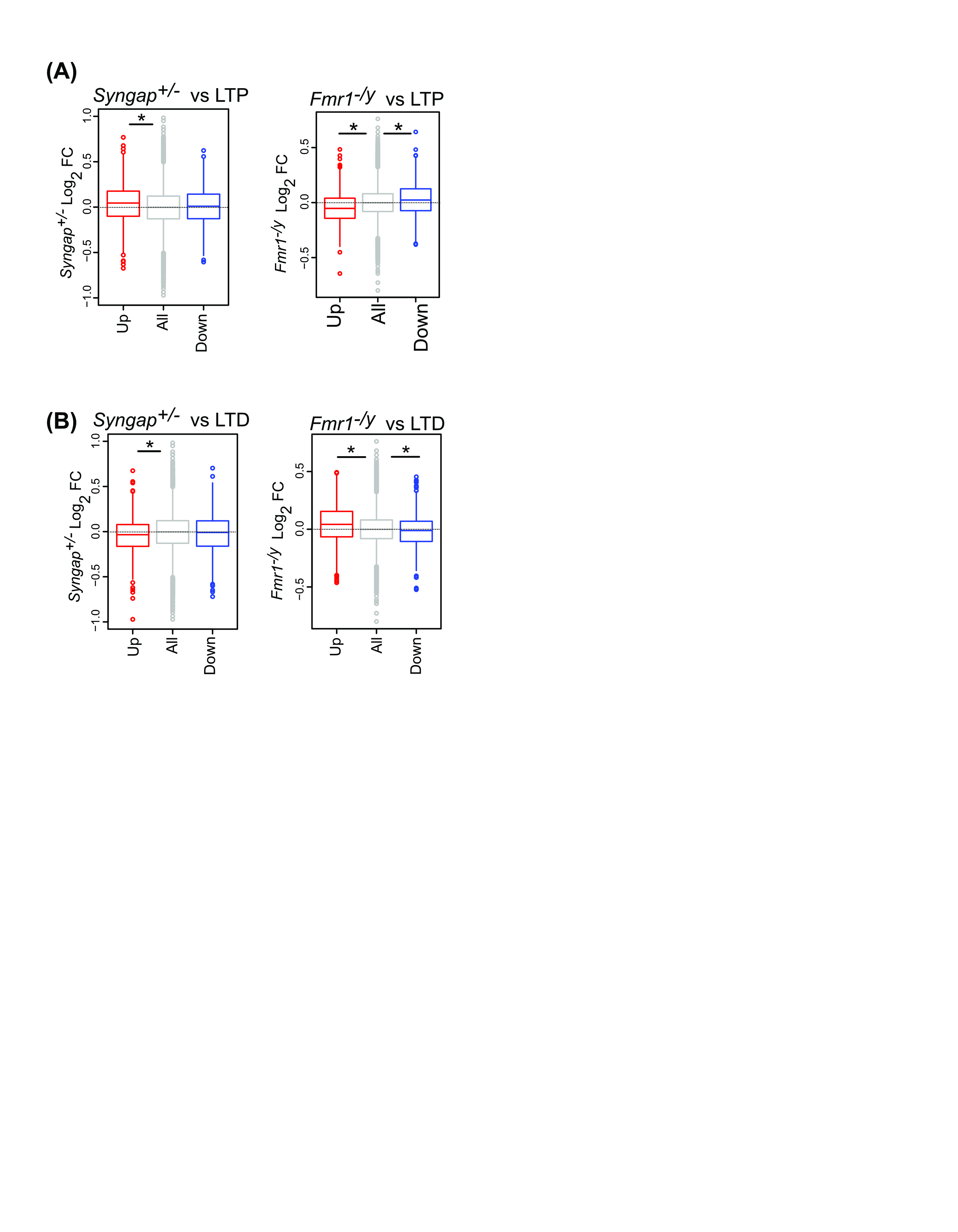

Supplement: Figure 2-2 — Comaprison of cLTP and mGLUR-LTD related translation changes in WT with Syngap+/- and Fmr1-/y CA1 neurons. (A) Analysis of the LTP significant transcripts in the Syngap+/- translatome shows significant increase in LTP- upregulated transcripts but no change in LTP- downregulated transcript (Kruskal-Wallis test *P = 3.30e-13, Post hoc two-sided Wilcoxon rank-sum test up *P = 4.77e-14, down P = 0.97), while LTP significant transcripts in the Fmr1-/y translatome show significant opposing change in both groups- LTP- upregulated and LTP- downregulated transcripts (Kruskal-Wallis test *P < 2.2e-16, Post hoc two-sided Wilcoxon rank-sum test up *P < 2.2e-16, down *P = 0.00013). (B) Analysis of the LTD significant transcripts in the Syngap+/- translatome shows significant decrease in LTD- upregulated transcripts (Kruskal-Wallis test *P = 9.81e-05, Post hoc two-sided Wilcoxon rank-sum test up *P = 2.29e-05, down P = 0.67). In contrast, LTD significant transcripts in the Fmr1-/y translatome show notably significant increase in LTD-upregulated transcripts (Kruskal-Wallis test *P =1.587e-11, Post hoc two-sided Wilcoxon rank-sum test up *P =7.71e-11, down *P = 0.0026). Boxplots display the distribution of Log2FoldChange values across LTP/LTD - up and down - regulated group of transcripts. The box represents the interquartile range (25th - 75th percentile), the center line indicates the median, and whiskers extend to 1.5 times the interquartile range. Data beyond the whiskers are shown as outliers. Download Figure 2-2, TIF file. [file eneuro-12-ENEURO.0086-25.2025-s009.tif]

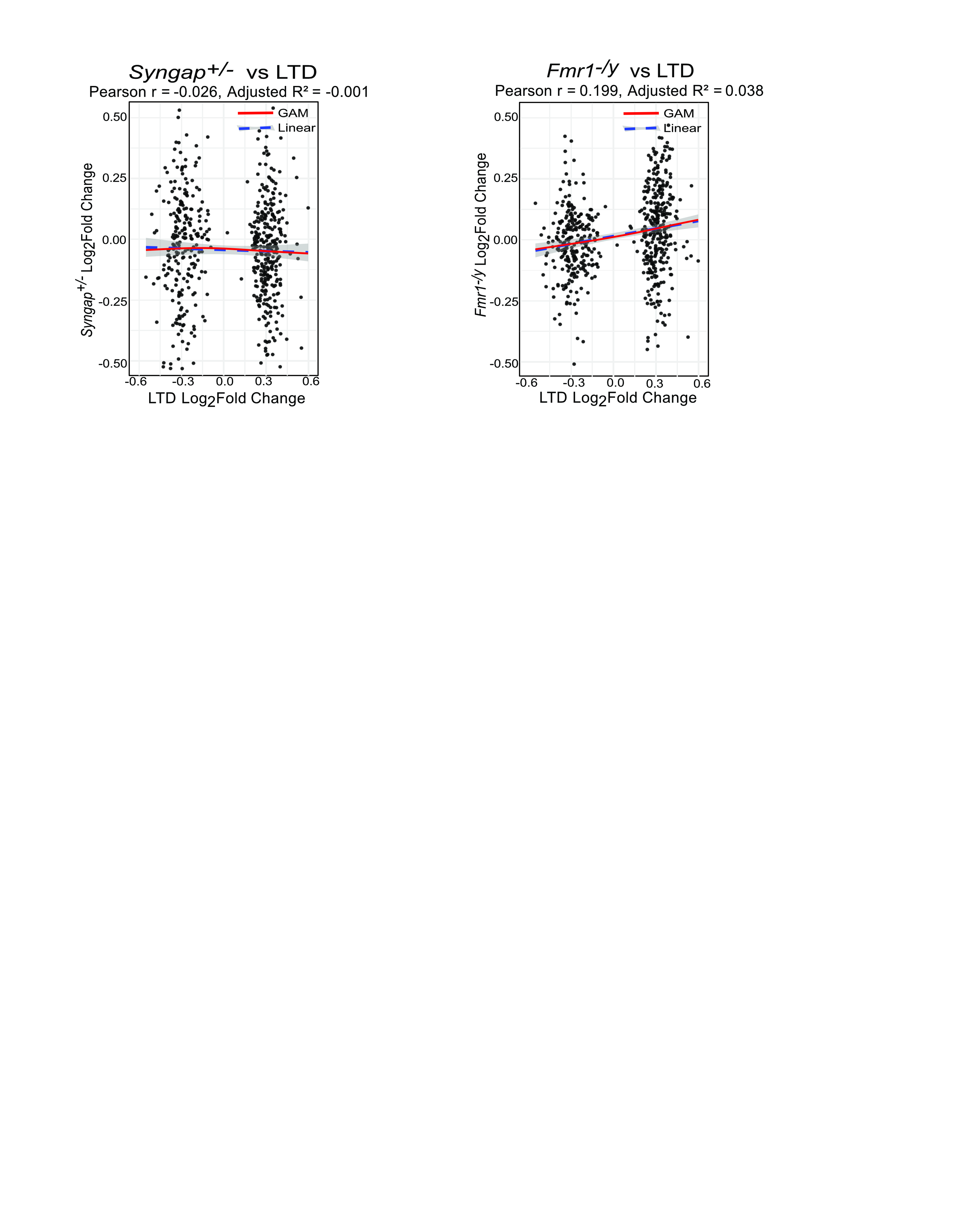

Supplement: Figure 3-1 — Non-linear modeling of the relationship between LTD-induced transcript changes and CA1-TRAP profiles in Syngap+/- and Fmr1-/y mice. Scatterplots show the log2 fold changes of transcripts significantly altered in the LTD dataset (P < 0.01) plotted against their corresponding fold changes in Syngap+/- (left) and Fmr1-/y (right) CA1-TRAP datasets. Linear regression fits (blue dashed lines) and Generalized Additive Model (GAM) fits (red curves) are overlaid. In the Fmr1-/y comparison, a small but significant positive correlation is observed, with close overlap between the GAM and linear fits, indicating a largely linear relationship. In contrast, no correlation was observed in the Syngap+/- dataset, and slight deviation of the GAM curve from the linear trend suggests a non-uniform or flat relationship between LTD-regulated genes and their expression in Syngap+/- neurons. Download Figure 3-1, TIF file. [file eneuro-12-ENEURO.0086-25.2025-s007.tif]

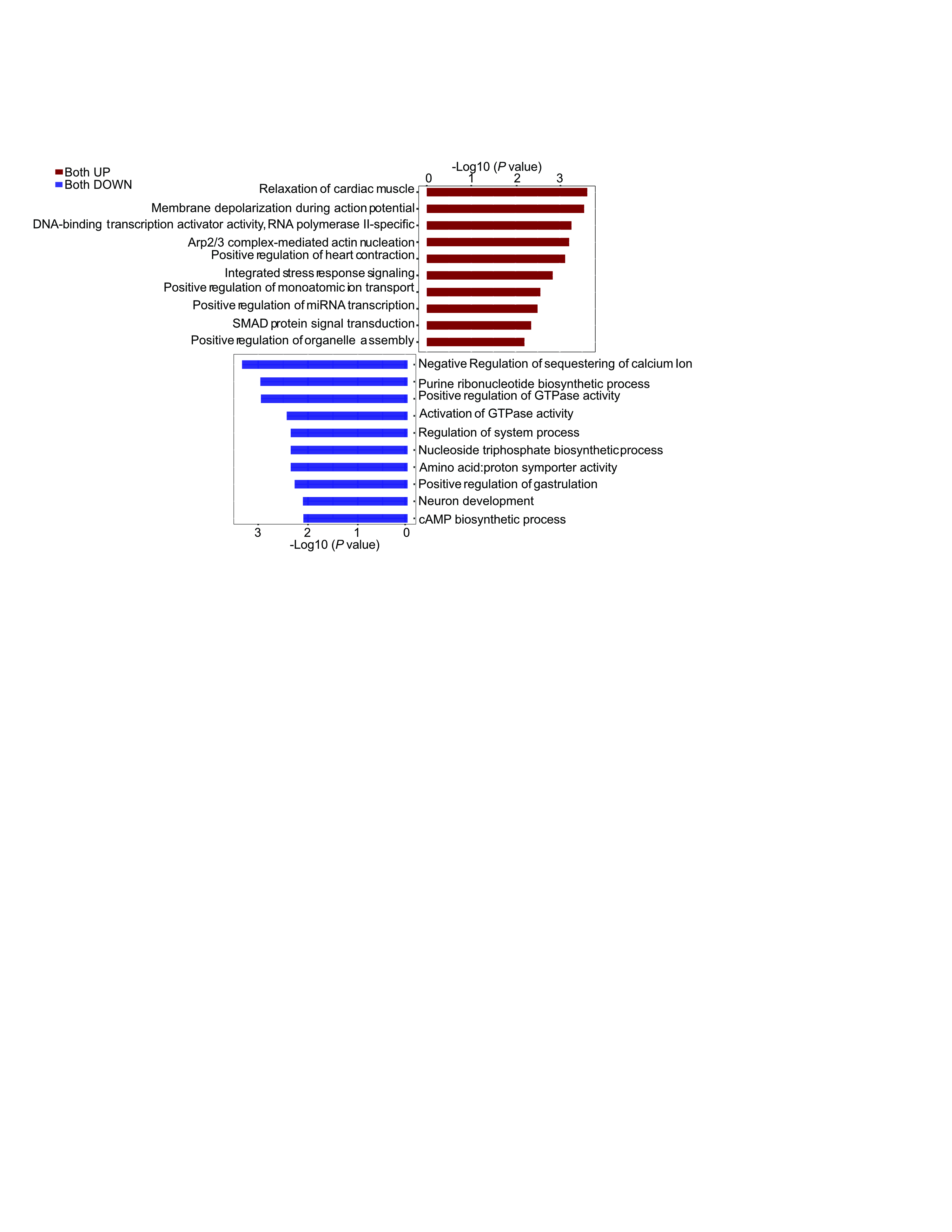

Supplement: Figure 4-1 — Gene ontology analysis of commonly changed transcripts in LTP and LTD. GO terms enriched in the overtranslated populations in both LTP & LTD regulate membrane depolarization, DNA binding while the commonly undertranslated transcripts primarily regulate calcium ion sequestration, GTPase activity, nucleotide biosynthesis. Download Figure 4-1, TIF file. [file eneuro-12-ENEURO.0086-25.2025-s010.tif]

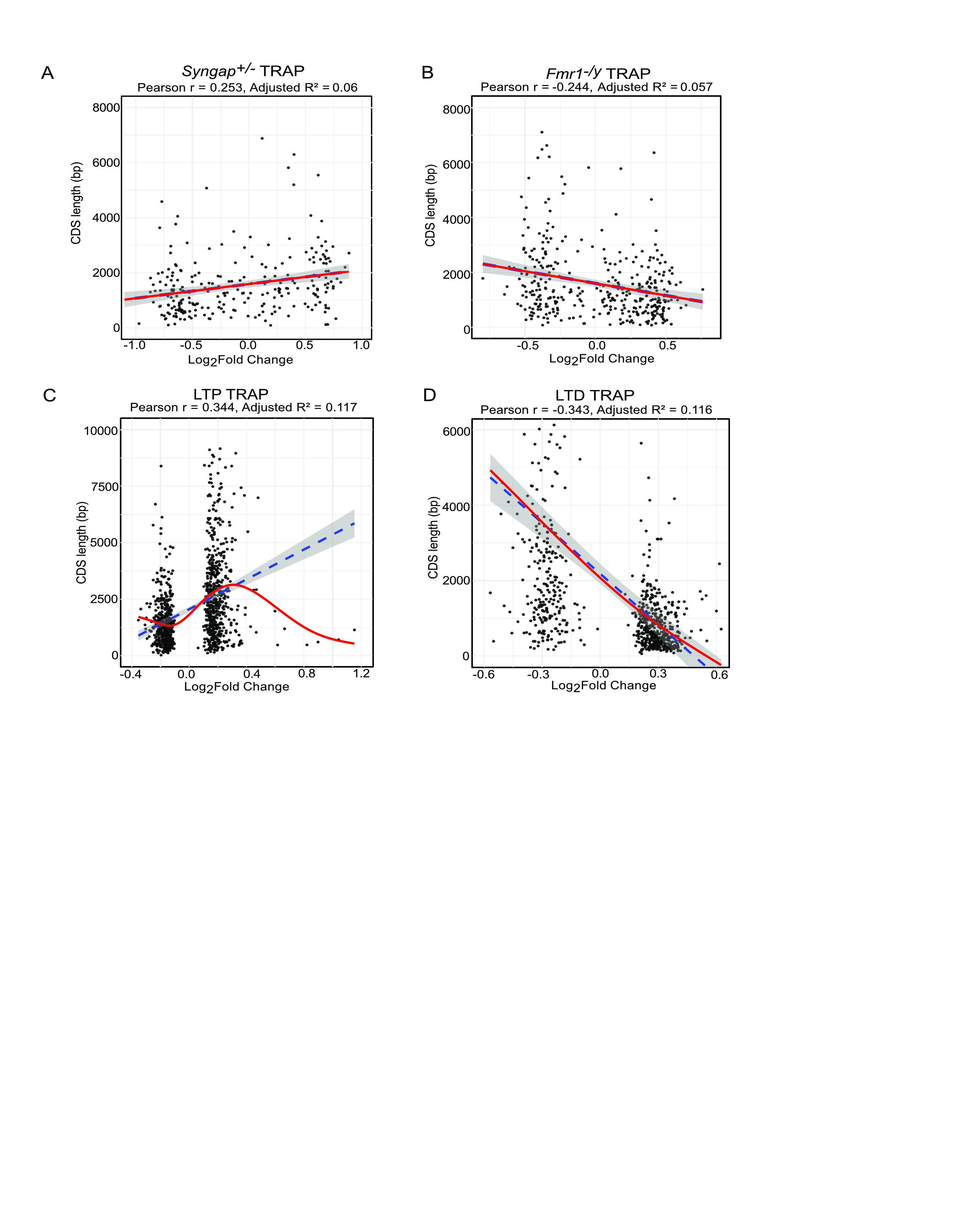

Supplement: Figure 5-1 — Non-linear modeling of the relationship between CDS length and transcript regulation in Syngap+/- and Fmr1-/y CA1-TRAP datasets and WT cLTP/LTD datasets. Scatterplots show the relationship between transcript coding sequence (CDS) length and log2 fold change in: (A) Syngap+/- CA1-TRAP vs LTP-induced genes, (B) Fmr1-/y CA1-TRAP vs LTD-induced genes, (C) WT CA1-TRAP following cLTP stimulation, and (D) WT CA1-TRAP following mGluR-LTD. Each panel displays a linear regression fit (blue dashed line) and a Generalized Additive Model (GAM) fit (red curve). In the WT cLTP and Syngap+/- datasets, longer CDS length was positively associated with transcript upregulation, though the GAM fits in Syngap+/- suggest a complex non-linear trend with peak upregulation observed in a subset of transcripts under ∼2500 bp. Conversely, in LTD and Fmr1-/y datasets, CDS length showed a negative correlation with transcript expression while the GAM fit indicate subtle deviations from linearity. Download Figure 5-1, TIF file. [file eneuro-12-ENEURO.0086-25.2025-s005.tif]

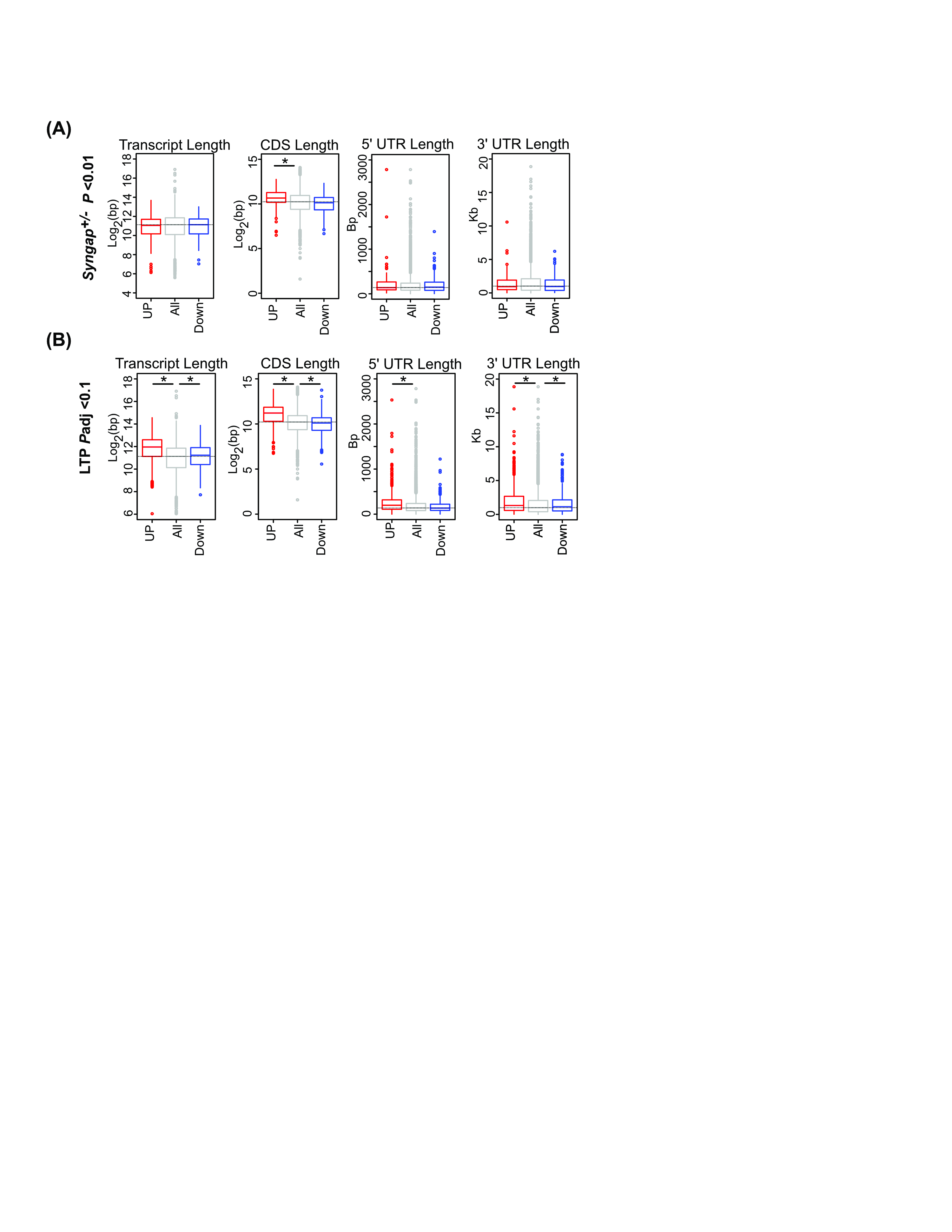

Supplement: Figure 5-2 — Genomic features analysis of significant transcripts (P value < 0.01) in Syngap+/- and cLTP datasets. (A) Analysis of the transcript length in Syngap+/- translatome (Kruskal-Wallis test P = 0.52); Analysis of the CDS length in Syngap+/- translatome (Kruskal-Wallis test *P = 0.0001, Post hoc two-sided Wilcoxon rank-sum test up *P = 3.80e-05, down P = 0.05); Analysis of the 5’ UTR length in Syngap+/- translatome (Kruskal-Wallis test P = 0.6362); Analysis of the 3’ UTR length in Syngap+/- translatome (Kruskal-Wallis test P = 0.88). (B) Analysis of the transcript length in LTP translatome (Kruskal-Wallis test *P < 2.2e-16, Post hoc two- sided Wilcoxon rank-sum test up *P < 2.2e-16, down *P = 0.017); Analysis of the CDS length in LTP translatome (Kruskal-Wallis test *P < 2.2e-16, Post hoc two-sided Wilcoxon rank-sum test up *P < 2.2e- 16, down *P = 0.002); Analysis of the 5’ UTR length in LTP translatome (Kruskal-Wallis test *P < 2.2e-16, Post hoc two-sided Wilcoxon rank-sum test up *P < 2.2e-16, down P = 0.15); Analysis of the 3’ UTR length in LTP translatome (Kruskal-Wallis test *P = 3.38e-09, Post hoc two-sided Wilcoxon rank-sum test up *P = 5.97e-10, down *P = 0.04). Boxplots display the distribution of Log2 base pairs (bp) for transcript length or cds length and bp or Kilo-bp values of UTR lengths across Up and Down - regulated group of transcripts. The box represents the interquartile range (25th - 75th percentile), the center line indicates the median, and whiskers extend to 1.5 times the interquartile range. Data beyond the whiskers are shown as outliers. Download Figure 5-2, TIF file. [file eneuro-12-ENEURO.0086-25.2025-s002.tif]
